# Supplementary material for: Therapeutic potential of oncolytic Zika virus in NF1-associated tumors
Source: Mol Ther Oncol. 2026 Oct 21;34(4):201370. doi: 10.1016/j.omton.2026.201370 (PMC13761349; doi:10.1016/j.omton.2026.201370)
Supplement: Supplementary file 1 [file mmc1.pdf]

OMTON, Volume *34*

Supplemental information

Therapeutic potential of oncolytic Zika virus in NF1-associated tumors

Raiane Oliveira Ferreira, Hui Ling Ma, Elisa Helena Farias Jandrey, Isabela Granha, Camila Correia Avelino, Gabriel Bandeira do Carmo, Amanda Assoni, Gustavo Sartorelli, Rafael Braga Petito, Rodrigo S. Moura-Neto, Rosane Silva, Hamilton Matushita, Oswaldo Keith Okamoto, Karin Soares Cunha, and Mayana Zatz

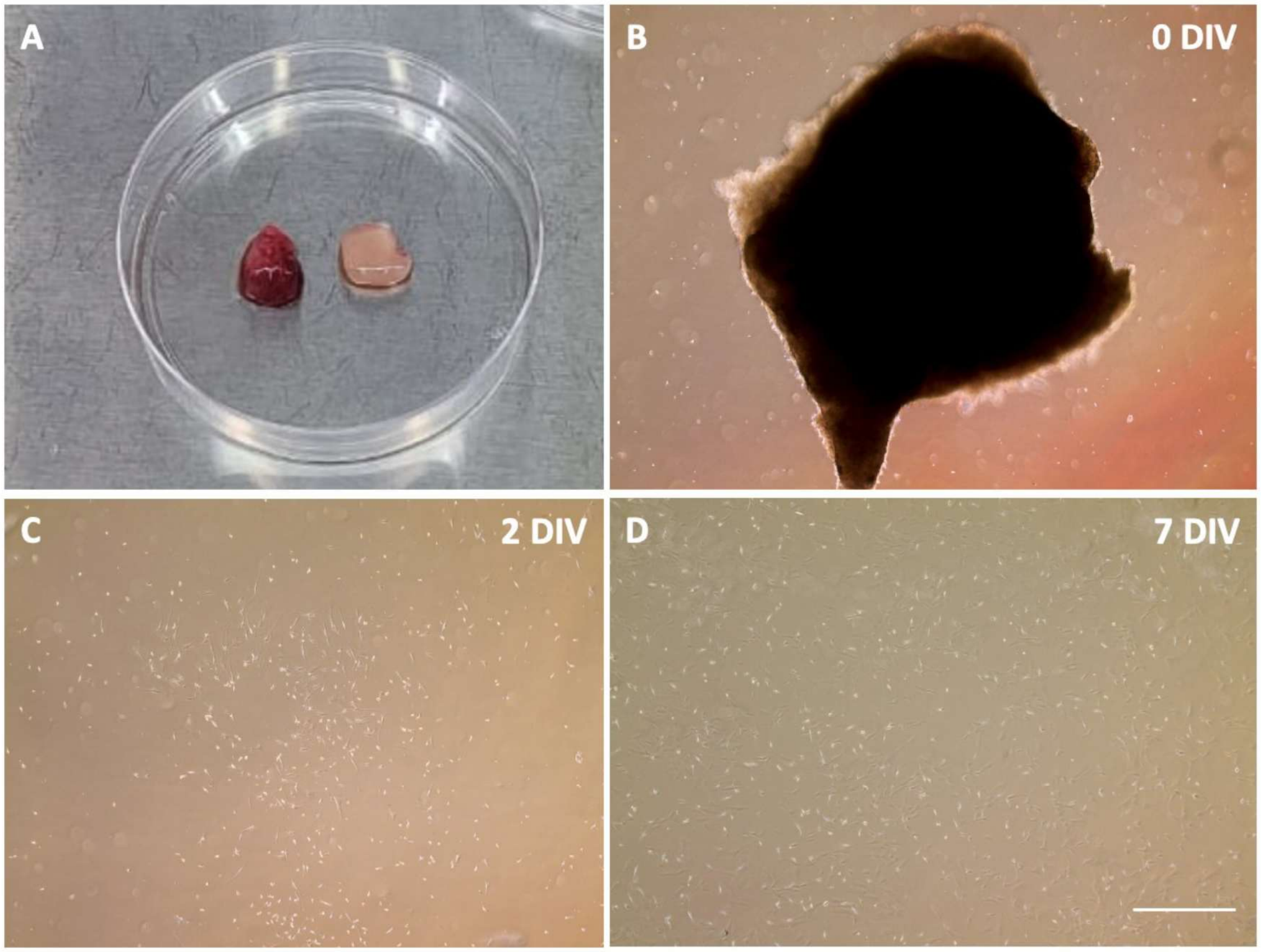

Figure S1. Cell isolation from patient-derived sample. A) Patient-derived fresh tumor tissue. Representative bright-field microscopy of the primary monolayer culture at 0 days *in vitro* (DIV) (B), 2 DIV (C), and 7 DIV (D). Scale bar: 200um.

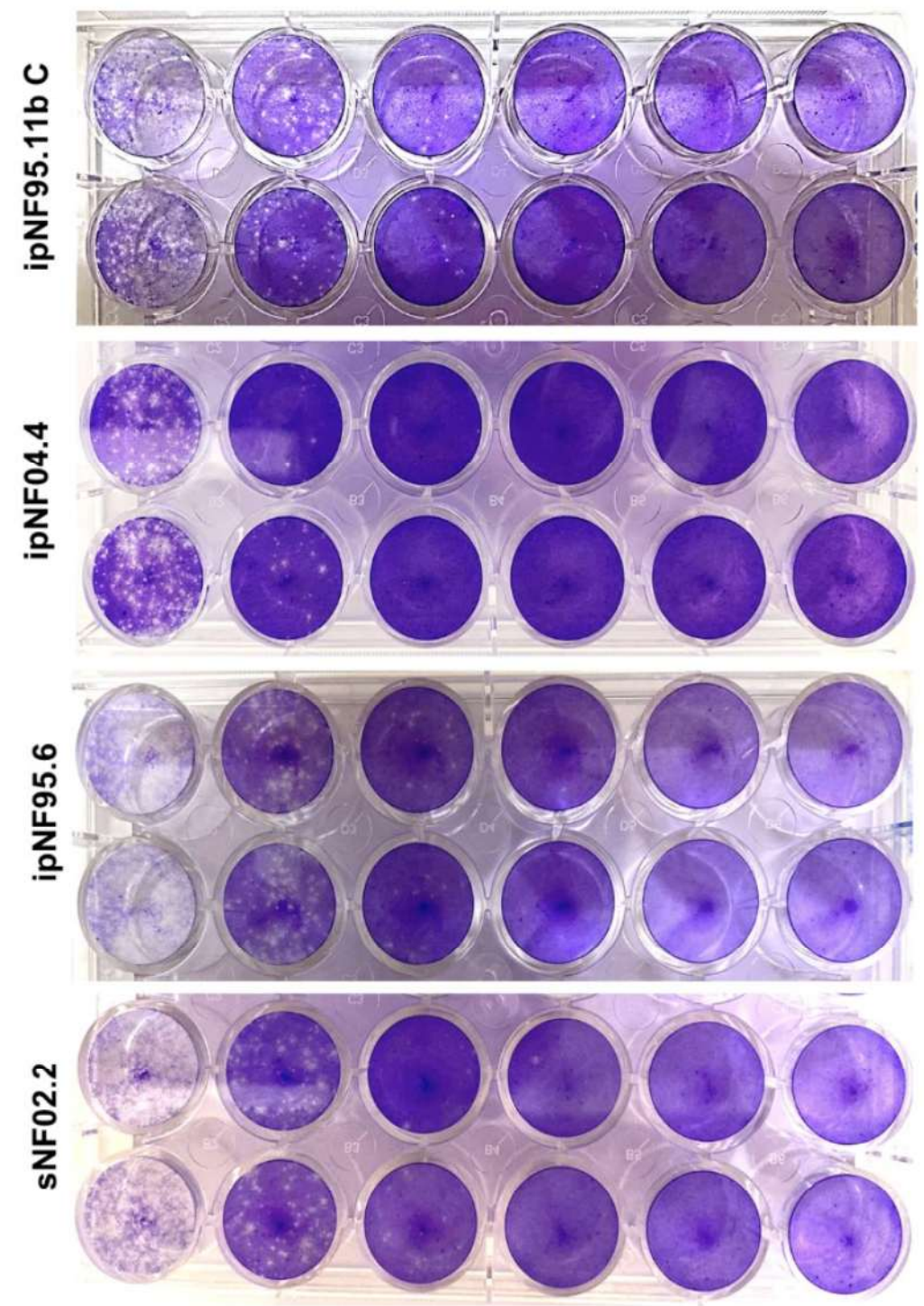

Figure S2. ZIKV replication in NF1 cells. To assess viral replication, PFU assays were measured in the supernatants collected from NF1 cells infected with ZIKV at MOI of 5, 48 h post-infection. A) Viral plaques were visualized and counted in VERO cells four days after infection. B) Quantification of infectious viral particles is expressed as Log PFU mL^{-1}. Significance was considered at *$p < 0.05$, **$p < 0.01$, ***$p < 0.001$, ****$p < 0.001$ and ns = not significant.

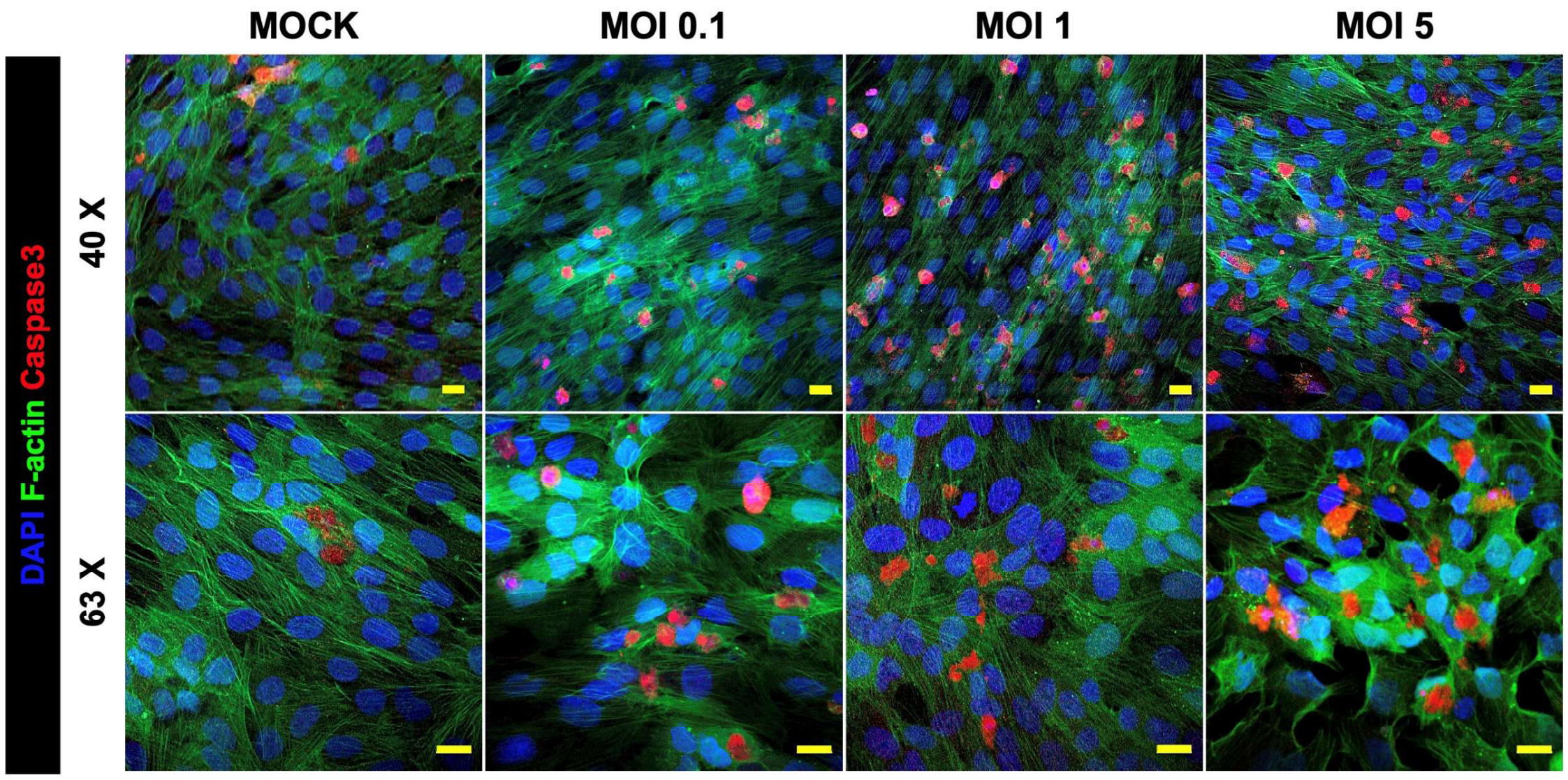

Figure S3. Immunofluorescence images of ipNF95.11b C 72hpi for clivage-caspase3 (red) and F-actin (green). Scale bar: 20um.

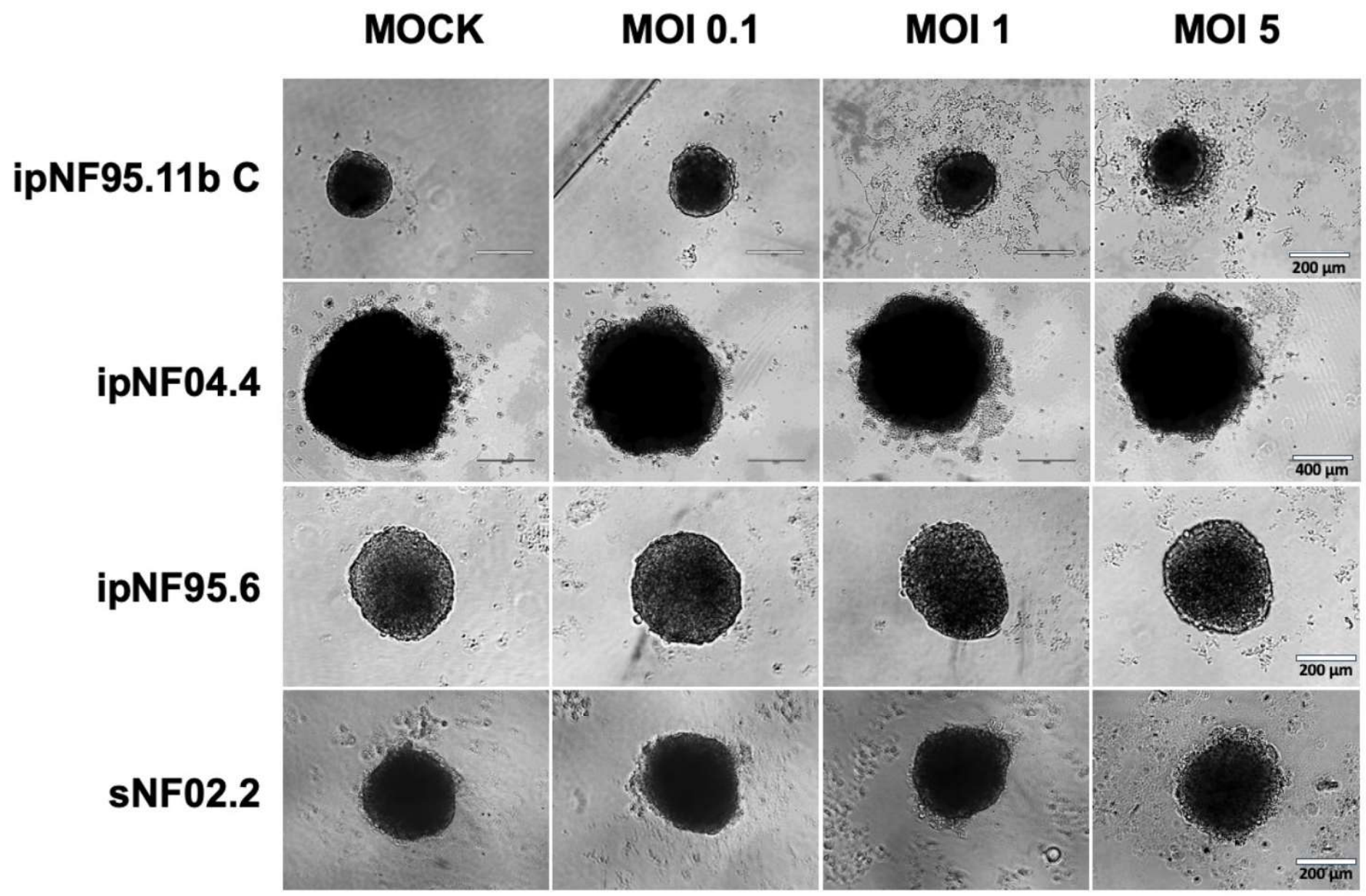

Figure S4. Bright-field images of 3D neurofibromatosis spheroid cultures, infected with ZIKV at MOCK, MOI 0.1, 1, and 5, were captured 72 hpi, revealing alterations in spheroid morphology and diameter changes.

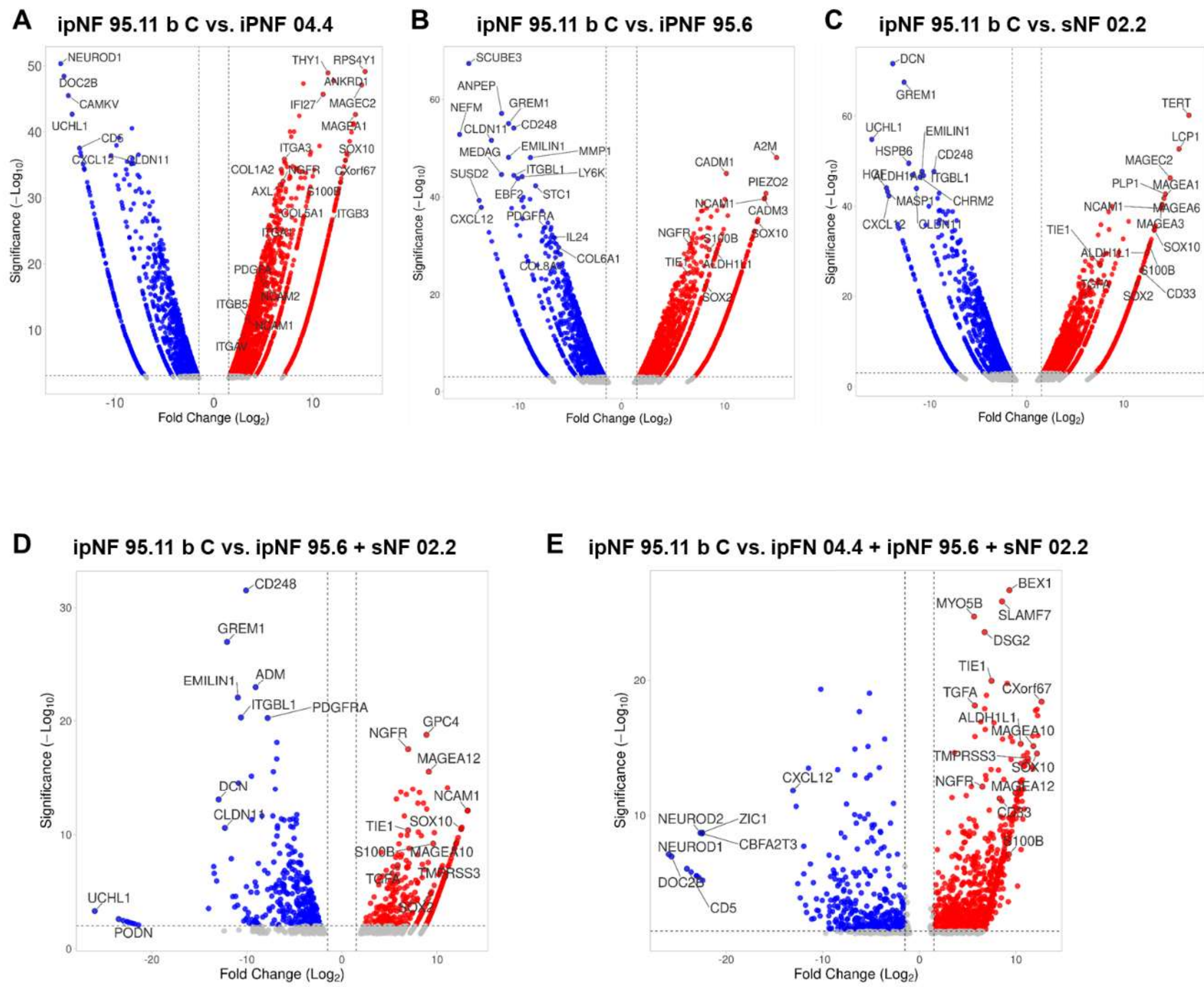

Figure S5. Differential gene-expression profiles across NF1 tumor cell line comparisons. Volcano plots depict $\log_2$ fold-change (x-axis) versus statistical significance ($-\log_{10}$ p-value, y-axis). Upregulated transcripts are shown in red, and downregulated transcripts in blue. Individual represent pairwise contrasts relative to the ipNF 95.11 b C cell line: A) vs. ipNF 04.4. B) vs. ipNF 95.6. C) vs. sNF 02.2. D) vs. ipNF 95.6 + sNF 02.2. E) vs. ipNF 04.4 + ipNF 95.6 + sNF 02.2.

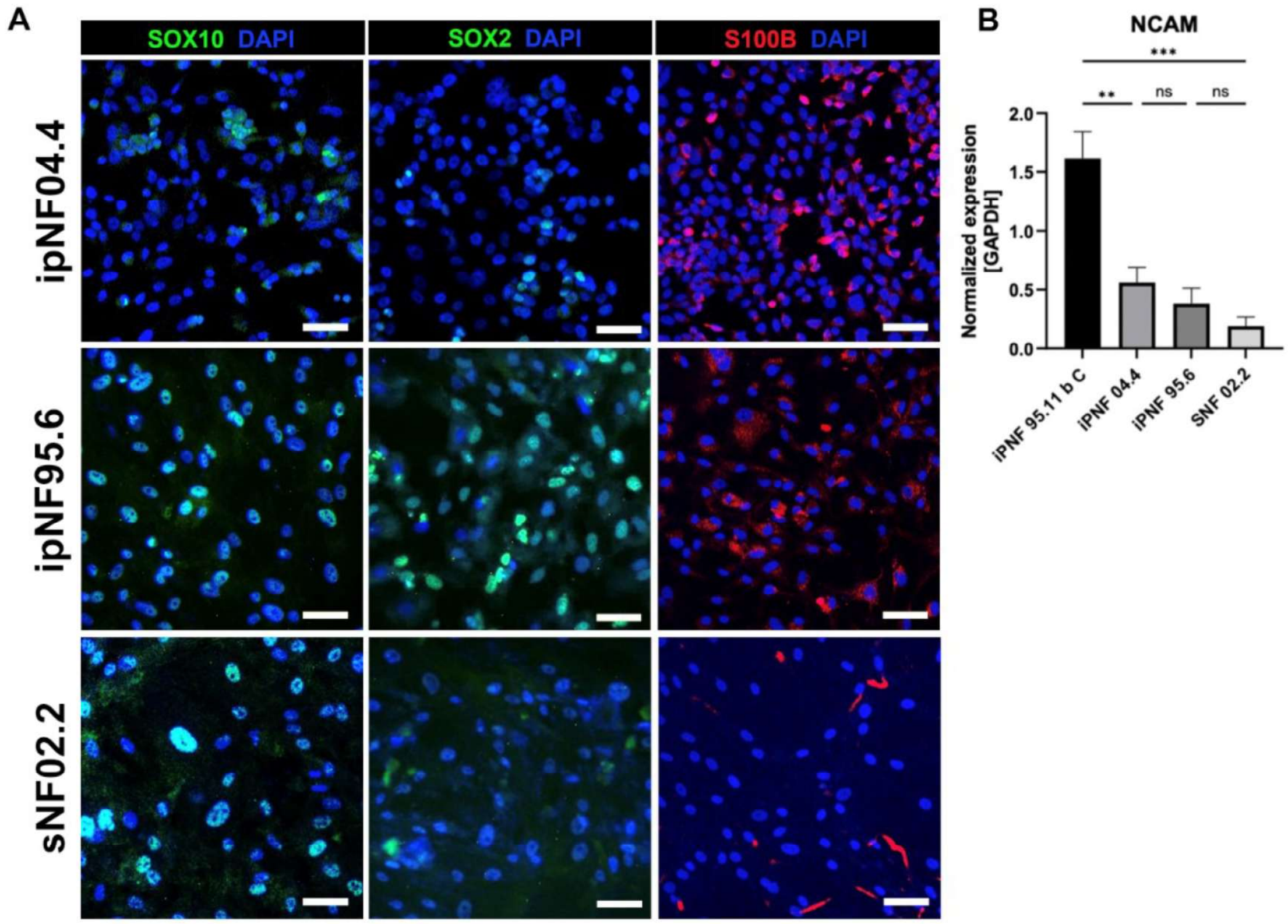

Figure S6. A) Immunofluorescence images of NF1 tumor cell lines for SOX10 (green), SOX2 (green), S100B (red), and nuclei (DAPI, blue). B) Real-time PCR results showing the relative expression levels of NCAM in NF1 tumor cell lines, normalized to GAPDH. Statistical significance: **$p < 0.01$, ***$p < 0.001$, ns = not significant.

Table S1. Primer sequence for RT-qPCR

Target	Sequence (5'- 3')
**GAPDH**	F: GCTGGAGGGCGAAGAAAAC
	R: GCCTTCTGACACGGATTTGG
**NCAM1**	F: CCGGAGAGTACATCTGCACC
	R: GAGCTTAGGTGCACTGGGTT
**NESTIN**	F: GGC GCA CCT CAA GAT GTC C
	R: CTT GGG GTC CTG AAA GCT
**S100B**	F: AGGAAGAGGATGTCTGAGCTG
	R: CCTCCGGGTTAGGGTCTACA
**SOX10**	F: GTTGACTGTTGCACCCACAC
	R: GAGTGGTAAGGCCTCCGATG
**SOX2**	F: TTCACATGTCCCAGCACTACCAGA
	R: TCACATGTGTGAGAGGGGCAGTGTGC

Table S2. Pathogenic variants identified in whole-exome sequencing analysis.

Cell line	Sequence Ontology	Germline NF1 mutation
ipNF95.11b C	frameshift_variant	c.1756delACTA (p.Thr586Valfs*18)
ipNF95.6	stop_gained<br>stop_gained	p.Arg816Ter/ p.Arg2237Ter
sNF02.2	stop_gained	p.Arg2237Ter
C30322	frameshift_variant	p.Val2229Leufs*15
