# Supplementary material for: Therapeutic potential of oncolytic Zika virus in NF1-associated tumors
Source: Mol Ther Oncol. 2026 Oct 21;34(4):201370. doi: 10.1016/j.omton.2026.201370 (PMC13761349; doi:10.1016/j.omton.2026.201370)
Supplement: Supplementary file 2 [file mmc3.pdf]

Molecular Therapy
Oncology
Original Article

Therapeutic potential of oncolytic Zika virus in NF1-associated tumors

Raiane Oliveira Ferreira,[1,7] Hui Ling Ma,[1,7] Elisa Helena Farias Jandrey,[1] Isabela Granha,[1] Camila Correia Avelino,[1] Gabriel Bandeira do Carmo,[2] Amanda Assoni,[1] Gustavo Sartorelli,[1] Rafael Braga Petito,[3] Rodrigo S. Moura-Neto,[4] Rosane Silva,[5] Hamilton Matushita,[6] Oswaldo Keith Okamoto,[1] Karin Soares Cunha,[3] and Mayana Zatz[1]

[1]Human Genome and Stem Cell Research Center, University of Sao Paulo, Sao Paulo, Brazil; [2]A. C. Camargo Cancer Center, Sao Paulo, Brazil; [3]Department of Pathology, School of Medicine, Federal Fluminense University, Rio de Janeiro, Brazil; [4]Instituto de Biofísica Carlos Chagas Filho, Federal University of Rio de Janeiro, Rio de Janeiro, Brazil; [5]Instituto de Biologia, Federal University of Rio de Janeiro, Rio de Janeiro, Brazil; [6]Department of Neurosurgery, Division of Pediatric Neurosurgery, University of Sao Paulo, Sao Paulo, Brazil

Neurofibromatosis type 1 (NF1) is an autosomal dominant disorder caused by pathogenic NF1 variants that drive aberrant Ras signaling and predispose individuals to tumors, including plexiform neurofibromas (PNFs) and malignant peripheral nerve sheath tumors (MPNSTs). These tumors remain challenging to treat: surgery is often unfeasible, MEK inhibitors provide only partial and temporary benefit, and no effective targeted therapies exist for MPNSTs. Here, we identify a previously unrecognized susceptibility of NF1-associated tumors to Zika virus (ZIKV). ZIKV efficiently infects Schwann cell-derived lines from PNFs and MPNSTs, activates viral replication, and triggers a robust pro-inflammatory cytokine response. Infection decreased viability by ∼60% in PNFs and ∼40% in MPNSTs and reduced Ki-67 expression, consistent with cytotoxic activity. Tumor spheroids exhibited pronounced structural disruption and increased cell death. Transcriptomic analysis linked ZIKV sensitivity to SOX10 and SOX2 expression in neural crest-like tumor cells. Most importantly, this finding was confirmed when fresh infected patient-derived samples were infected with ZIKV. These findings provide the first evidence that ZIKV can kill NF1-associated tumor cells and highlight its potential as a novel therapeutic strategy for PNFs and MPNSTs.

INTRODUCTION

Neurofibromatosis type 1 (NF1) is a common autosomal dominant disorder affecting ∼1 in 2,500–3,500 individuals worldwide.[1] It is caused by heterozygous pathogenic variants in the *NF1* gene (chromosome 17q11.2), which encodes the tumor suppressor neurofibromin.[2] *NF1* exhibits one of the highest *de novo* mutation rates among human disease genes, roughly 1 in 10,000 gametes per generation, reflecting the gene's large size and complex genomic architecture.[3] Consistent with the absence of mutational hotspots, more than 4,400 pathogenic variants distributed across coding and non-coding regions have been cataloged in the Human Gene Mutation Database (HGMD).[4] Loss of neurofibromin in NF1 leads to constitutive Ras activation and downstream hyper-signaling through the Raf-MEK-ERK and PI3K-Akt-mTOR pathways,[5] driving abnormal cell proliferation and predisposing affected individuals to a spectrum of benign and malignant tumors, including neurofibromas, plexiform neurofibromas (PNFs), malignant peripheral nerve sheath tumors (MPNSTs), and optic pathway gliomas.[6]

PNFs occur in ∼50% of individuals with NF1 and are a major source of morbidity. Although histologically benign, they grow along nerve sheaths, infiltrate surrounding tissues, and frequently cause disfigurement, chronic pain, neurological deficits, and functional impairment.[7] Complete surgical removal is rarely feasible due to their location, size, and multifocality.[8] MEK inhibitors such as selumetinib and mirdametinib have recently been approved for symptomatic, inoperable PNFs in both pediatric and adult patients; however, responses are typically partial (≈30%–40% shrinkage), resistance commonly emerges, and treatment-limiting toxicities remain a concern.[9–11] As a result, effective long-term medical therapies are still lacking.

NF1 mutations in PNFs and MPNSTs disrupt Schwann cell maturation and maintain tumor cells in a neural-crest-like, stem-like state characterized by expression of SOX10, ErbB3, Nestin, and related markers, as demonstrated by single-cell studies in human and murine models.[12] This developmental vulnerability is particularly relevant in the context of oncolytic Zika virus (ZIKV) therapy. Although ZIKV, a flavivirus, typically causes mild or asymptomatic infection in adults, it exhibits strong tropism for neural progenitor cells (NPCs). In the context of oncology, this neurotropism has been successfully exploited against central nervous system malignancies; human induced pluripotent stem cell (iPSC)-derived forebrain organoids and glioblastoma models consistently demonstrate that ZIKV targets and eliminates

Received 19 March 2026; accepted 20 October 2026;
https://doi.org/10.1016/j.omton.2026.201370.

[7]These authors contributed equally

Correspondence: Mayana Zatz, Human Genome and Stem Cell Research Center, University of Sao Paulo, Brazil.
E-mail: mayazatz@usp.br

© 2026 The Authors. Published by Elsevier Inc. on behalf of The American Society of Gene and Cell Therapy.
This is an open access article under the CC BY license (http://creativecommons.org/licenses/by/4.0/).

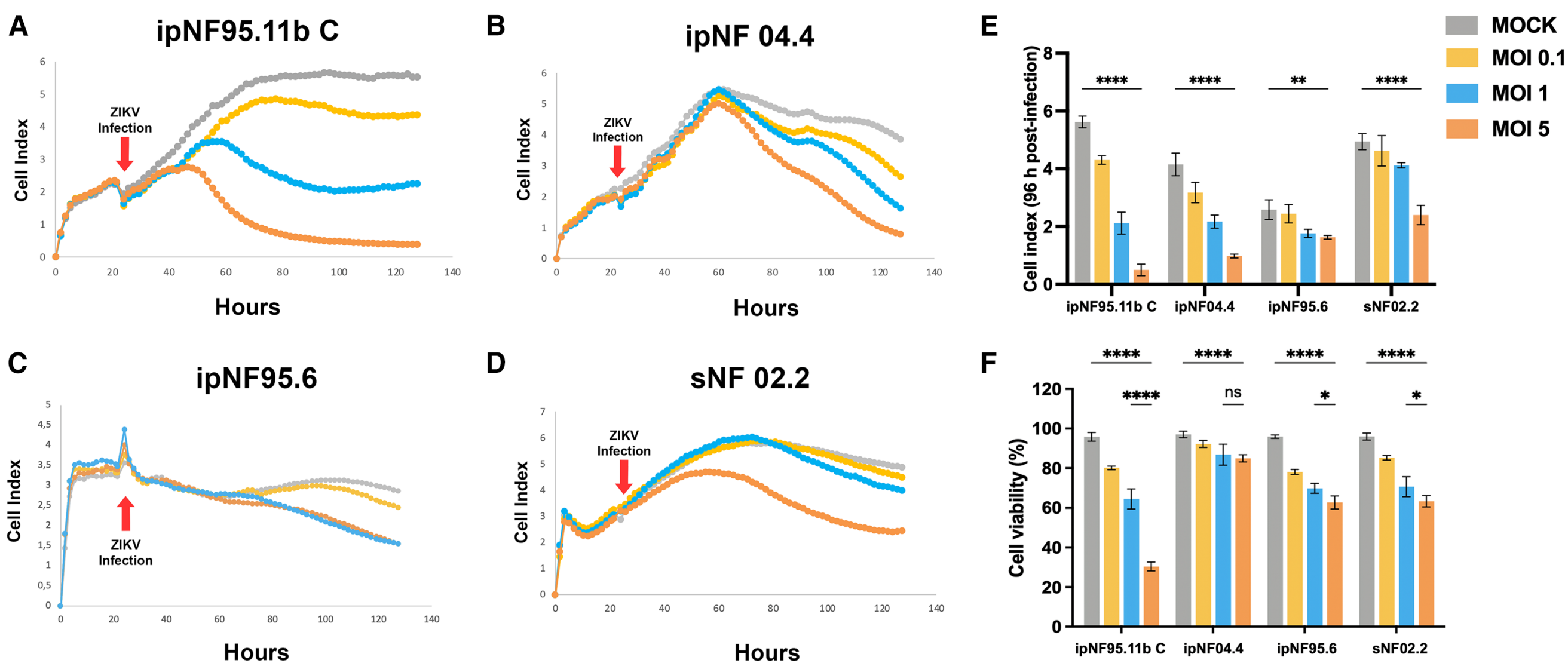

Figure 1. Cell proliferation and viability of NF1 tumor cell lines post-infection of ZIKV at mock and MOIs 0.1, 1, and 5

(A–D) Cell index plot with the real-time monitoring xCELLigence system. (E) Cell index at 96 hpi. (F) MTT cell viability analysis was performed at 72 hpi. Significance was considered at *$p < 0.05$, **$p < 0.01$, and ****$p < 0.0001$; ns, not significant.

neural cancer stem-like cells (CSCs) primarily through SOX2-dependent mechanisms, while sparing mature neural populations.[13–16] Furthermore, preclinical studies in murine and canine brain-tumor models have shown robust oncolytic efficacy and safety.[17,18] Given the limited therapeutic options for NF1-associated tumors and the complete absence of studies examining ZIKV's effects on peripheral nerve-derived neoplasms, we sought to investigate whether ZIKV possesses oncolytic activity against NF1-associated tumor cells.

RESULTS

Genetic characterization of NF1-derived tumor cell lines

All NF1 tumor cell lines harbored at least one pathogenic loss-of-function (LoF) variant in the *NF1* gene, including three stop-gain and one frameshift (Table S2). In the ipNF95.6, we identified two *NF1* heterozygous pathogenic variants, while in ipNF95.11b C, one *NF1* homozygous pathogenic variant was detected. These findings are consistent with somatic second-hit events caused by intragenic mutation and loss of heterozygosity, respectively.[19] In the ipNF04.4 and sNF02.2, only one heterozygous pathogenic variant was detected. Additionally, in ipNF95.11b C we identified one heterozygous pathogenic missense variant in EGFR, a well-known oncogene with a role in MPNST tumorigenesis.[20]

ZIKV infects NF1-derived tumor cell lines and inhibits proliferation

To interrogate the effects of ZIKV on NF1-derived tumors, four different cell lines were infected. All cultures were susceptible to ZIKV cytotoxicity, with the ipNF95.11b C displaying the highest sensitivity, showing a pronounced reduction in viability at 96 hours post-infection (hpi) (Figures 1A–1D). The cell index values (Figure 1E) and MTT assays (Figure 1F) support these findings.

NF1 tumor cells express ZIKV receptors, are susceptible to infection, and support viral replication

We further investigated whether NF1 tumors are susceptible to ZIKV and examined the expression of potential viral receptors. At an MOI of 5, over 45% of ipNF95.6 and sNF02.2 were infected, while infection reached nearly 80% in the ipNF95.11b C (Figures 2A and 2B). Moreover, infection rates increased with higher MOIs (*$p < 0.05$), demonstrating a clear dose-dependent relationship between viral inoculum and infection efficiency.

All evaluated cell lines were permissive to ZIKV replication (Figures 2C and S2). Among them, sNF02.2 produced the highest levels of infectious virus (1.82×10^5 PFU/mL).

We analyzed the expression of ZIKV entry receptors AXL and ITGAV (Figures 2D and 2E). AXL was present in ipNF95.11b C, ipNF95.6, and sNF02.2, with no significant differences in expression levels among them (Figure 2F). ITGAV expression was also observed, with ipNF95.11b C showing significantly higher levels compared with the others (**$p < 0.01$) (Figure 2G). These findings suggest that higher AXL and ITGAV expression may be associated with increased susceptibility to ZIKV infection in NF1 tumor cells. Notably, the ipNF04.4 cell line lacking even detectable AXL and ITGAV protein remained permissive to ZIKV.

ZIKV infection induces apoptosis and reduces proliferation in NF1 tumor cell lines

Caspase-3/7 activation at an MOI of 0.1 was minimal across all cell lines except for ipNF95.11bC, which showed a modest increase (Figures 3A and 3B). Marked apoptosis occurred at MOIs of 1 and 5, most prominently in ipNF95.11b C, which exhibited

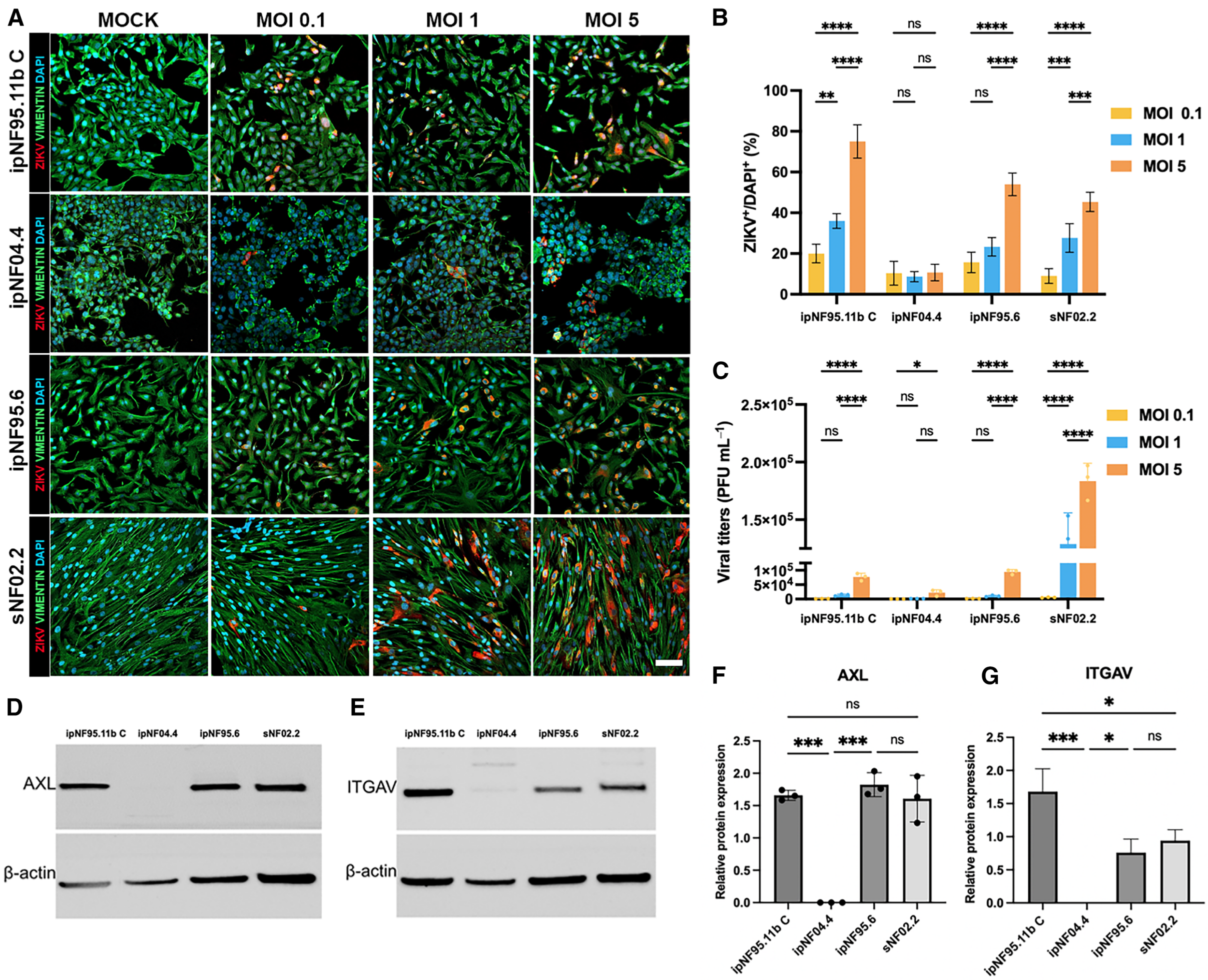

Figure 2. ZIKV infection and replication on NF1 tumor cell lines

(A) Immunostaining of NF1-derived cell lines infected with ZIKV at MOIs of 0.1, 1, and 5 for 48 hpi. Nuclei (DAPI, blue), ZIKV detected with anti-NS1 (red), and vimentin (green). Scale bars, 100 μm. (B) Percentage of ZIKV-positive cells relative to DAPI-positive nuclei on the basis of immunofluorescence image analysis. (C) Quantification of infectious viral particles expressed as Log PFU mL^{-1} in supernatants collected from NF1-derived tumor cell lines infected with ZIKV at MOIs of 0.1, 1, and 5 for 48 hpi. (D and E) Western blots showing ZIKV receptors AXL and ITGAV. (F and G) Quantification of relative protein AXL and ITGAV expression normalized to β-actin. Significance was considered at $^{*}p < 0.05$, $^{**}p < 0.01$, $^{***}p < 0.001$, and $^{****}p < 0.0001$; ns, not significant.

approximately 10-fold and 30-fold increases in caspase-positive cells, respectively. Consistently, Figure S3 confirms this apoptotic phenotype by demonstrating cleaved caspase-3 activation alongside preserved plasma membrane integrity. In contrast, sNF02.2 showed very low caspase-3/7 staining even at MOI 5, suggesting that ZIKV-induced cell death in MPNST cells may proceed through caspase-independent pathways or occur later than 72 hpi.

To determine whether ZIKV affected cell cycle activity, we quantified Ki-67, a standard marker of cell proliferation[21] (Figure 3C). Ki-67 levels were reduced in ZIKV-infected ipNF95.11b C, with a moderate decrease observed in ipNF95.6 and sNF02.2 (Figure 3D). In contrast, ipNF04.4 maintained high Ki-67+ at this time point. These findings suggest that ZIKV-mediated growth inhibition in NF1 tumor cells is associated with reduced Ki-67 expression.

ZIKV exhibits cytotoxicity in 3D NF1 tumor spheroids

To establish a representative NF1 model, tumor spheroids were generated (Figure 4A). Each cell line formed spheroids with distinct morphologies and diameters, reflecting intrinsic differences in aggregation behavior and suggesting diverse microenvironmental conditions (Figure S3). Although spheroids were generally more resistant to ZIKV than 2D cultures, ZIKV exposure still caused a significant decrease in viability and spheroid size (Figures 4B–4D). Consistent with our monolayer results, ipNF95.11b C remained

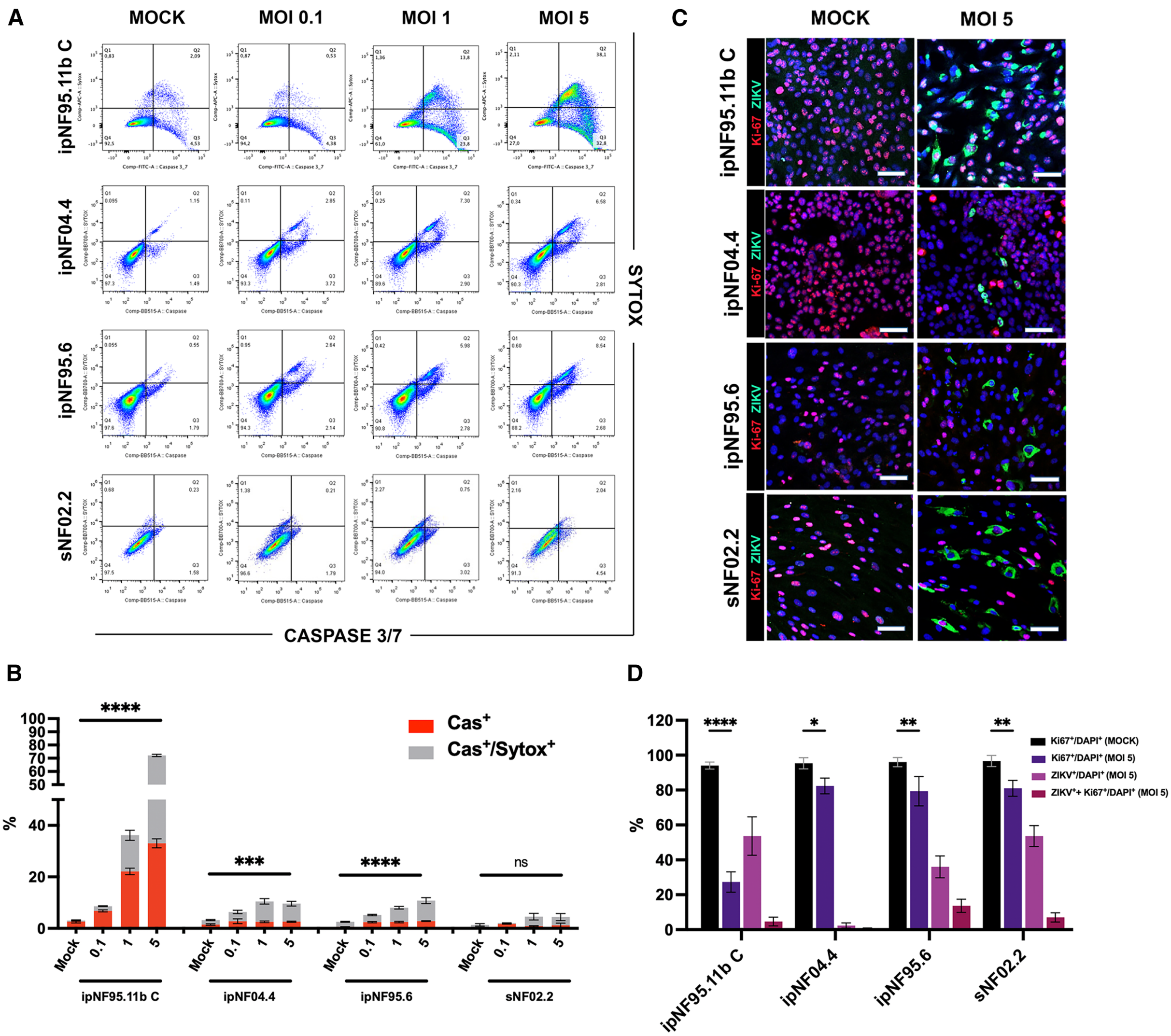

Figure 3. ZIKV induces caspase-mediated cell death and impacts Ki67 expression in NF1-derived tumor cells

(A) Cells were labeled with the CellEvent caspase-3/7 and SYTOX AADvanced Dead Cell Stain, and analyzed by flow cytometry at 72 hpi. (B) Quantification of cell death profiles showing the percentage of caspase-3/7$^+$/SYTOX$^+$ double-positive cells (necrotic) and caspase-3/7$^+$/SYTOX$^-$ single-positive cells (apoptotic). (C) Confocal images showing ZIKV (green), Ki-67 (red), and nuclei (DAPI, blue) at 48 hpi. Scale bars, 50 μm. (D) Quantification of ZIKV$^+$, Ki-67$^+$, and ZIKV$^+$/Ki-67$^+$ double-positive cells, expressed as a percentage of total DAPI-stained nuclei. Statistical significance indicates differences between the Ki-67$^+$/DAPI$^+$ (MOCK) and Ki-67$^+$/DAPI$^+$ (MOI 5) groups, with *$p < 0.05$, **$p < 0.01$, ***$p < 0.001$, and ****$p < 0.0001$; ns, not significant.

the most susceptible, whereas ipNF04.4 showed comparatively higher resistance under identical conditions.

ZIKV leads to upregulation of pro-inflammatory cytokines in NF1 tumor cells

We analyzed the immunological responses of NF1 tumor cells following ZIKV infection. Heatmap profiling revealed distinct cytokine and chemokine expression patterns across the groups (Figure 5A). Principal component analysis (PCA) further demonstrated clear separation among them, with ipNF95.11b C and sNF02.2 exhibiting the strongest responses (Figure 5B). ipNF95.11b C displayed a strong and broad pro-inflammatory response, marked by high secretion of multiple immunomodulatory mediators (Figure 5C). sNF02.2 exhibited a moderate but diverse induction of inflammatory factors, whereas ipNF95.6 showed an intermediate response. In contrast, ipNF04.4 demonstrated minimal cytokine induction, consistent with its low susceptibility to ZIKV.

Network analysis of the secreted mediators (Figure 5D) revealed dense cytokine-chemokine interaction networks in ipNF95.11b C and sNF02.2, highlighting coordinated signaling involving IL-1

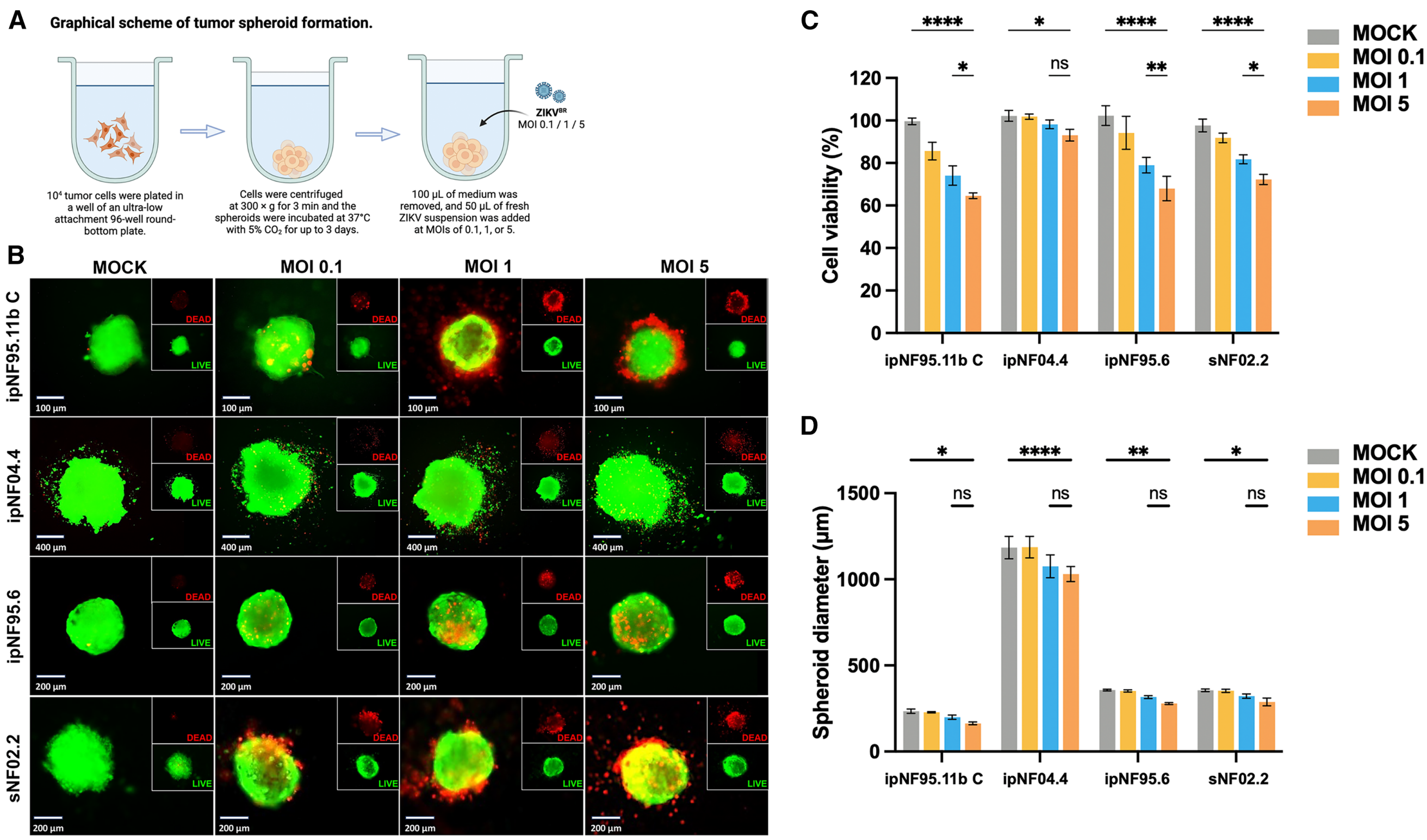

Figure 4. ZIKV infection in 3D NF1-derived spheroid cultures

(A) Graphical scheme of the tumor spheroid formation methodology. (B) Live/dead staining was performed at 72 hpi, live (green) and dead (red) cells. (C) Cell viability of spheroids was assessed with the PrestoBlue assay. (D) Spheroid diameters were measured at 72 hpi. Significance was considered at *$p < 0.05$, **$p < 0.01$, and ****$p < 0.0001$; ns, not significant.

family members, interferons, chemokines such as CCL3/4 and CXCL8/10, and growth factors including CSF3. Enrichment analysis confirmed significant overrepresentation of pathways related to inflammatory responses, immune activation, and T cell signaling.

The ZIKV cytotoxicity effect correlates with specific gene profiles of NF1 tumor cell lines

To elucidate the molecular basis for the variations in ZIKV susceptibility seen in our functional assays, we analyzed the baseline transcriptomic profiles of the four cell lines. As shown by RNA sequencing (RNA-seq) data, the lines exhibited a clear transcriptional segregation (Figures 6A–6C). These results demonstrate that each NF1 tumor cell line carries a distinct molecular signature before infection, which may underlie their variable susceptibility and inflammatory responses to ZIKV.

The transcriptomic heatmap further highlighted these differences (Figure 6D). ipNF95.11b C displayed the most divergent profile, marked by a broad cluster of highly upregulated genes and a smaller set of downregulated transcripts.

We generated volcano plots of differentially expressed genes (DEGs) across NF1 tumor cell line comparisons on the basis of their susceptibility to ZIKV infection and cell death (Figure S5). The hierarchical overlap of DEGs across multiple comparisons with ipNF95.11b C as the reference reveals a consistent set of strongly upregulated genes, including SOX10, S100B, and NGFR, indicating a shared transcriptional program characteristic of neural crest-derived Schwann cell lineages (Figure 6E). In contrast, genes such as CXCL12, RASGRF2, and RRAGA remain consistently downregulated, suggesting repression of chemokine signaling and Ras-related pathways. The Sankey plot links individual genes to their most associated human cell-type signatures and highlights prominent enrichment for neural crest-related lineages (Figure 6F). Markers including SOX10, S100B, PLP1, NGFR, FOXD3, PAX3, ALDH1L1, NEUROD2, and ZIC1 map primarily to Schwann cells, neural crest cells, oligodendrocytes, and neurons, reflecting their developmental origins. Additional genes associate with mesenchymal and cancer stem cell signatures, underscoring the heterogeneous cellular programs active in these tumors.

Immunofluorescence confirmed the Schwann cell-like identity predicted for ipNF95.11b C, which displayed abundant SOX10+ and SOX2+ nuclei and intense S100B staining (Figure 6G). Quantitative

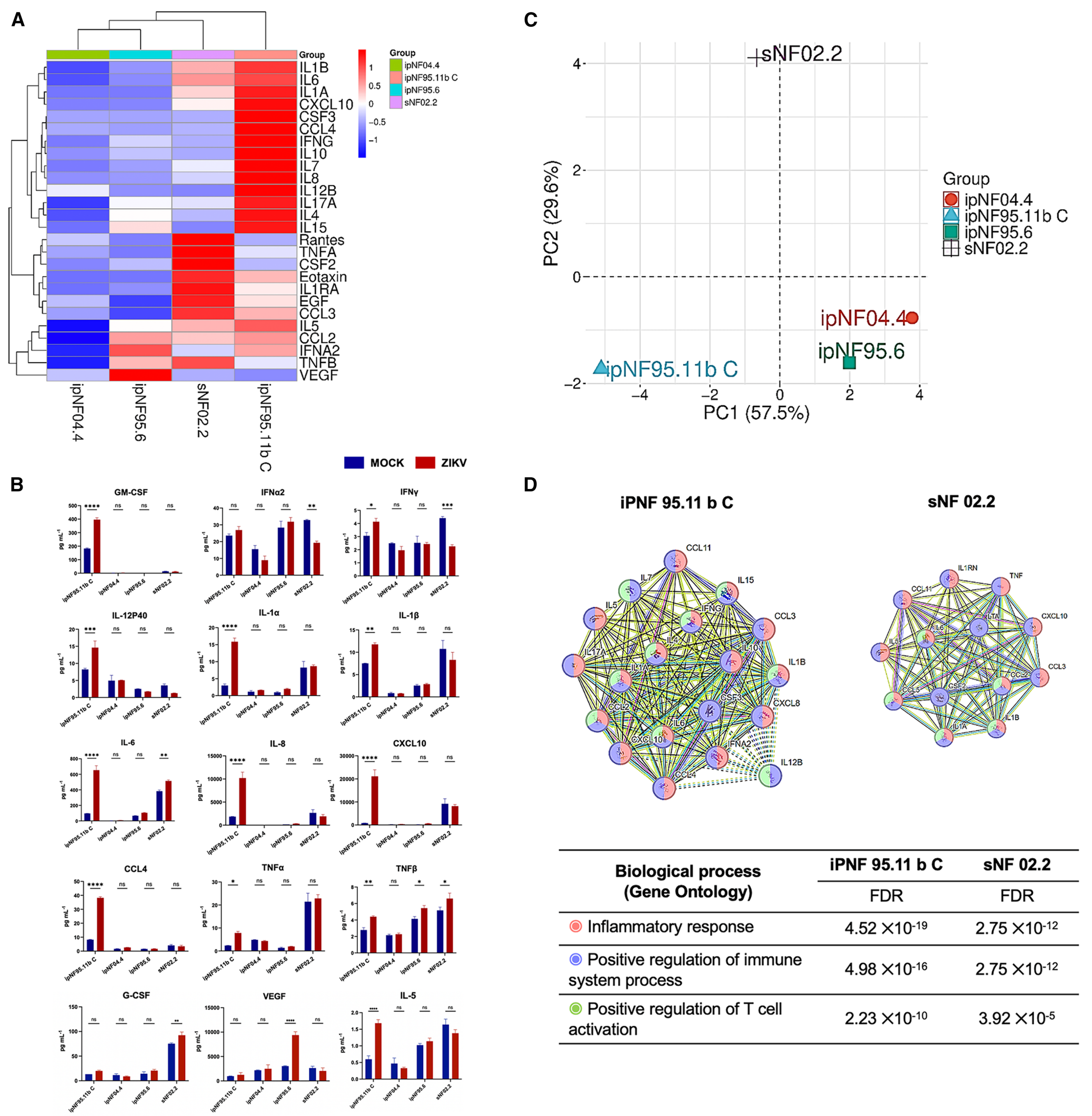

Biological process (Gene Ontology)	iPNF 95.11 b C FDR	sNF 02.2 FDR
Inflammatory response	4.52×10^{-19}	2.75×10^{-12}
Positive regulation of immune system process	4.98×10^{-16}	2.75×10^{-12}
Positive regulation of T cell activation	2.23×10^{-10}	3.92×10^{-5}

Figure 5. ZIKV triggers cell type-specific cytokine/chemokine responses in NF1 tumor cells 48 hpi with MOI 1

(A) Heatmap of cytokine/chemokine expression in ZIKV-infected ipNF95.11b C, ipNF95.6, and sNF02.2 cells. (B) Quantification of cytokine/chemokines from supernatants 48 hpi. (C) PCA plot showing distinct clustering by cell type. (D) Protein-protein interaction network with enriched pathways related to inflammatory response, positive regulation of immune system process, and T cell activation. Significance was considered at *$p < 0.05$, **$p < 0.01$, ***$p < 0.001$, and ****$p < 0.0001$; ns, not significant.

PCR (qPCR) analysis further supported these results (Figures 6H–6J). NESTIN and NCAM expression was also highest in ipNF95.11b C (Figures 6K and S6). These findings confirm that ipNF95.11b C retains a Schwann cell-like, progenitor-associated identity with high SOX2/SOX10 levels, likely contributing to its increased susceptibility to ZIKV.

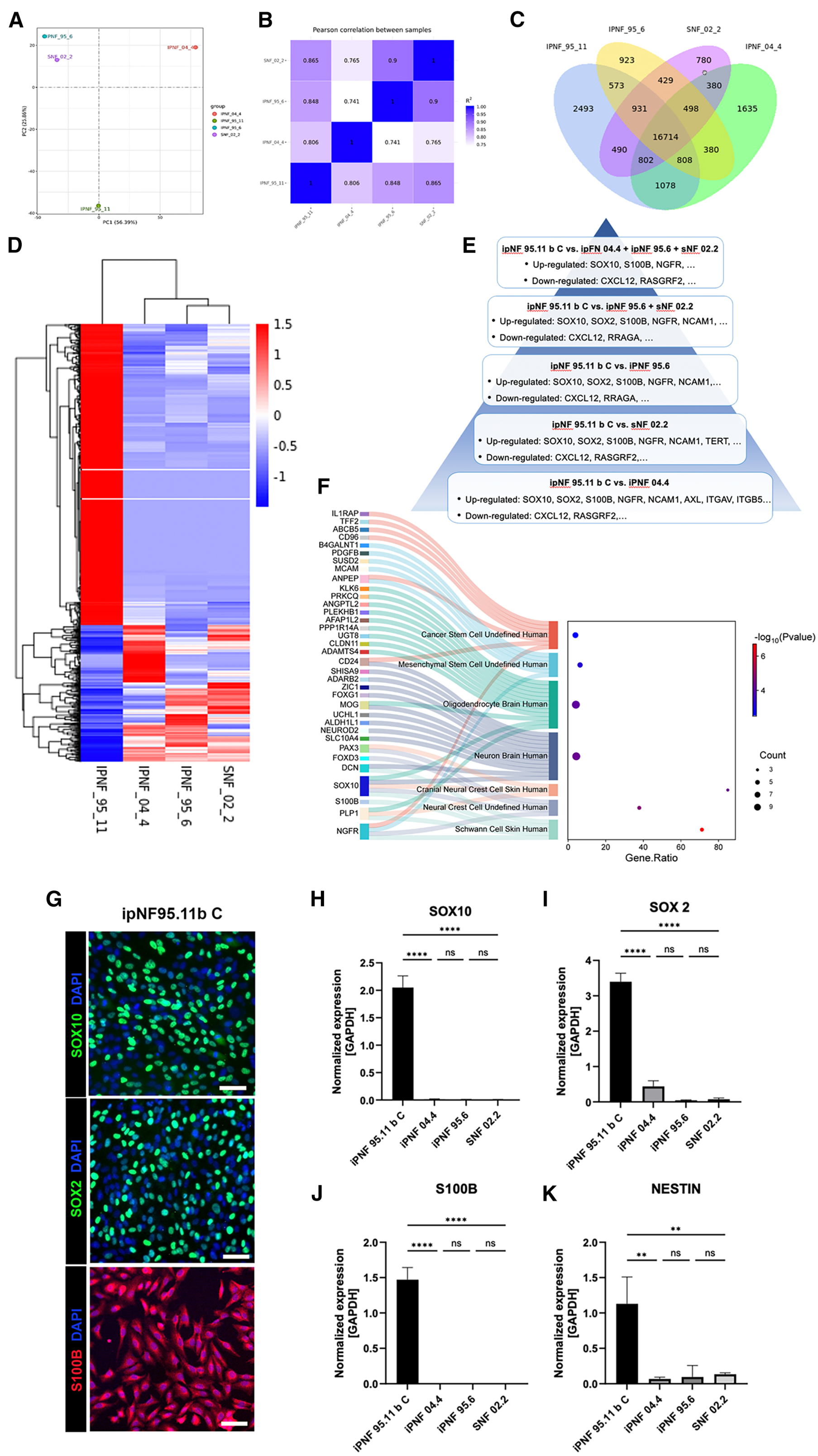

(legend on next page)

SOX10+/SOX2+ patient-derived tumor cells display high susceptibility to ZIKV

To validate our *in vitro* findings in a clinically relevant and translational context, we next assessed ZIKV infection in freshly derived patient tumor cells (C30322) harboring a frameshift NF1 variant (p.Val2229Leufs15) (Table S2). This primary culture exhibited SOX10 and SOX2 expression levels comparable to those observed in the highly susceptible ipNF95.11b C cell line (Figures 7A–7C), and expressed key Schwann cell- and ZIKV-associated markers, including S100B, NCAM, AXL, and ITGAV (Figures 7D–7G). Notably, at 72 hpi, patient-derived tumor cells were highly permissive to ZIKV, showing a marked reduction in viability even at a low multiplicity of infection (MOI = 0.1) (Figures 7H–7J). Together, these data extend our cell line-based observations to primary patient material and reinforce the notion that SOX10 and SOX2 expression is associated with increased susceptibility to ZIKV infection in NF1-associated tumors.

DISCUSSION

We report for the first time that ZIKV efficiently infects NF1 tumor cells, exerting dose-dependent cytotoxicity across both 2D and 3D culture platforms. Although cytotoxicity assays indicate that 3D NF1 models display higher baseline resistance than 2D monolayers, this is consistent with the reduced permissiveness of spheroid cultures to viral infection, attributed to the compact multicellular architecture that restricts viral diffusion and limits access to target cells.[22] Since ZIKV relies on ongoing active replication to propagate through dense tumor structures, achieving enhanced clearance in 3D environments may simply depend on an increased viral dose or a prolonged post-infection time frame. This observation aligns with our previous findings demonstrating that ZIKV possesses a unique capacity to target and infiltrate 3D glioblastoma cultures, ultimately driving robust immune activation and programmed cell death.[23]

The well-established ZIKV entry receptors AXL and ITGAV[24] were consistently expressed in the NF1 tumor cells most susceptible to ZIKV, indicating that these cells remain permissive to ZIKV via canonical receptor-mediated entry pathways. Moreover, all tested cells also supported robust MOI-dependent viral replication. This efficient intracellular replication is essential for oncolytic virotherapy, as it exploits tumor-cell-intrinsic defects in antiviral immunity, triggers selective cell lysis, releases progeny virions, and propagates infection to adjacent malignant cells, thereby amplifying therapeutic efficacy.[25]

ZIKV is known to orchestrate a multifaceted cell death landscape wherein endoplasmic reticulum (ER) stress, pyroptosis, and lytic programs can coexist alongside canonical apoptosis.[26] In NF1 cells, ZIKV predominantly activates caspase-3 and caspase-7, driving a programmed cell death cascade characterized by the release of immunogenic mediators. This profile constitutes a hallmark of immunogenic cell death, which possesses the potential to robustly enhance anti-tumor immunity.[27] An exception was observed in sNF02.2, which secreted high levels of G-CSF, a cytokine previously shown to suppress caspase-3 activation in Schwann cells.[28] This may partially account for the limited caspase-dependent cell death in this cell line despite its high infection rate and robust viral replication in our study. These results are consistent with the known susceptibility of NPCs to ZIKV-induced apoptosis during fetal neurodevelopment,[29] suggesting that NF1 tumor cells retain similar viral vulnerabilities.

ZIKV infection also upregulated a broad panel of inflammatory cytokines and chemokines in NF1 tumor cells, supporting a dual mechanism of oncolysis: direct induction of apoptosis and remodeling of the tumor microenvironment to enhance anti-tumor immunity. Increased expression of cytokines such as IL-2, IL-12, IL-15, and IFN-γ can potentiate $CD8^+$ T cell and NK cell cytotoxicity,[30,31] while chemokines like CXCL10 facilitate their recruitment to the tumor, fostering innate immune activation and promoting neutrophil infiltration.[32] Prior studies similarly reported that ZIKV induces cytokines such as IFN-γ and TNF-α, which increase tumor immunogenicity and sensitize cancer cells to apoptosis.[33] Together, these immune-stimulatory signals may help convert immunologically "cold" tumors into "hot" tumors with greater immune infiltration and responsiveness. ZIKV has demonstrated comparable oncolytic and immunomodulatory effects in other central nervous system tumors,[34,35] highlighting its potential therapeutic relevance for NF1-associated neoplasms.

The baseline transcriptomic profile of NF1 tumor cells revealed features that may predispose ipNF95.11b C to ZIKV-mediated cytotoxicity, consistent with the virus's tropism for neural cells. Neural crest-associated genes such as SOX10, SOX2, S100B, NGFR, and NCAM1 were upregulated, underscoring the pronounced neural

Figure 6. ZIKV preferentially infects $SOX10^+$ and $SOX2^+$ NF1 tumor cell lines

(A) PCA of baseline RNA-seq profiles showing distinct clustering of the four NF1 tumor cell lines prior to ZIKV exposure. PC1 separates ipNF04.4 from the other cell lines, while PC2 distinguishes ipNF95.11bC. (B) Pearson correlation matrix illustrating pairwise transcriptional similarity across samples. (C) Venn diagram depicting the overlap of expressed genes among the four cell lines. (D) Hierarchical clustering heatmap of differentially expressed genes, highlighting major transcriptional differences, particularly the unique expression profile of ipNF95.11bC. (E) Summary of major upregulated and downregulated genes in pairwise comparisons between ipNF95.11b C and each of the other NF1 tumor cell lines. (F) Sankey diagram mapping key differentially expressed genes to enriched cell-type signatures, indicating lineage-related transcriptional programs, and GO enrichment analysis of upregulated genes in ipNF95.11bC, showing overrepresentation of pathways related to neural development, stemness, and cellular signaling. (G) Immunofluorescence images of NF1 tumor cell lines for SOX10 (green), SOX2 (green), S100B (red), and nuclei (DAPI, blue). Scale bars, 50 μm. (H–K) Real-time PCR results showing the relative expression levels of SOX10, SOX2, S100B, and NESTIN in NF1 tumor cell lines, normalized to GAPDH. Statistical significance: $^{**}p < 0.01$, $^{****}p < 0.0001$; ns, not significant.

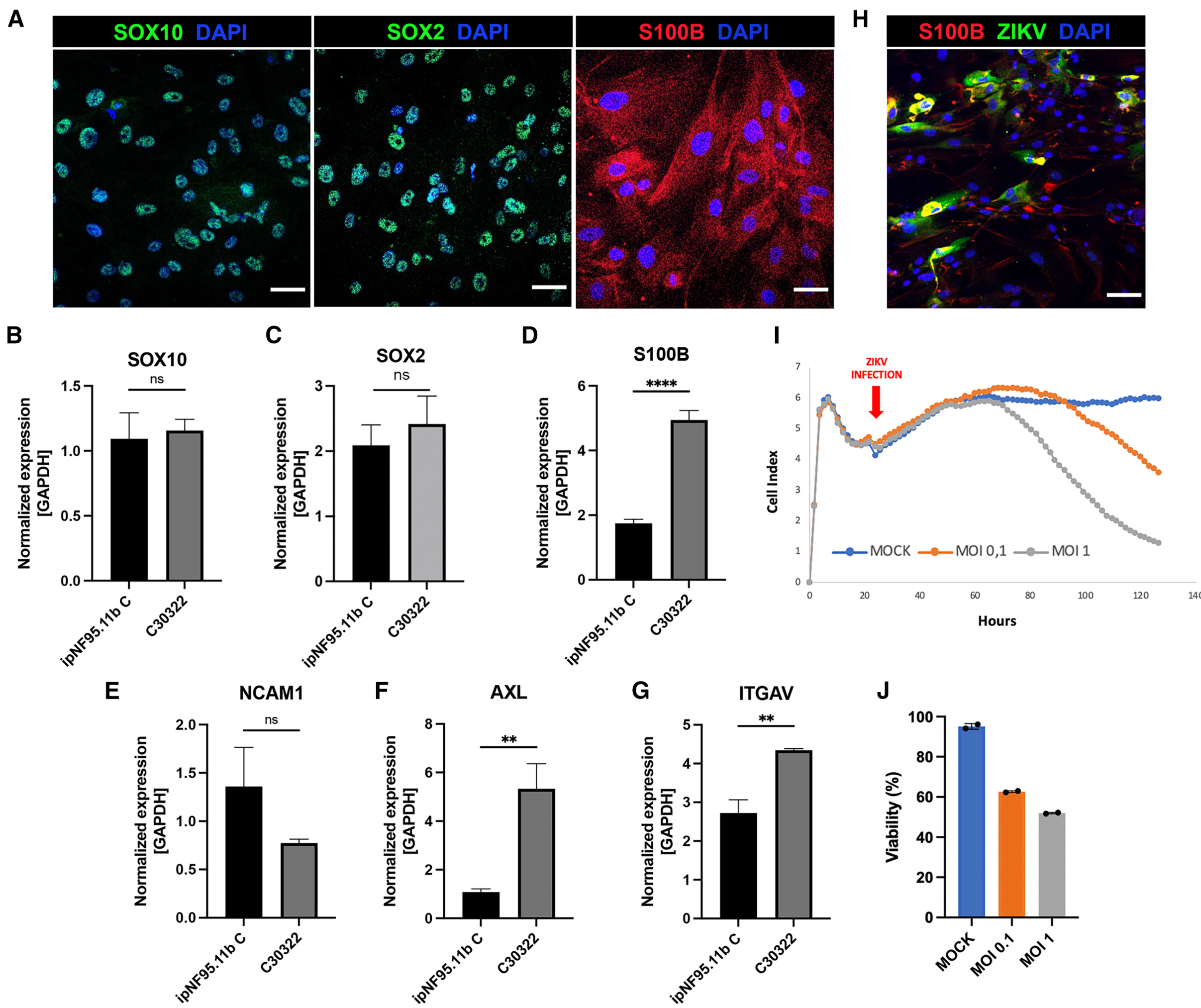

Figure 7. ZIKV effects in patient-derived primary tumor cell culture C30322

(A) Immunofluorescence images of patient-derived cells for SOX10 (green), SOX2 (green), S100B (red), and nuclei (DAPI, blue). (B–G) Real-time PCR results showing the relative expression levels of SOX10, SOX2, S100B, NCAM1, AXL, and ITGAV, normalized to GAPDH. Immunofluorescence images of patient-derived cells for S100B (red), ZIKV (green), and nuclei (DAPI, blue). (I) Cell index plot using the real-time monitoring xCELLigence system. (J) MTT cell viability analysis performed at 72 hpi. Statistical significance: ** $p < 0.01$, **** $p < 0.0001$; ns, not significant. Scale bars, 50 μm.

crest-derived identity of ipNF95.11b C.[12] The highly sensitive ipNF95.11b C lineage showed markedly stronger SOX10 and SOX2 staining, whereas the others exhibited only low expression. These observations point to a potential association between ZIKV cytotoxicity and SOX-family transcriptional programs. NF1 loss is known to drive neurofibroma formation by blocking Schwann cell differentiation and maintaining a SOX10^{+} progenitor-like state.[36] Neurofibromas consistently express SOX10, reflecting their Schwann cell origin, whereas SOX10 expression is often reduced or lost in MPNSTs, a hallmark of malignant dedifferentiation.[37]

Several antiviral defense genes were expressed at low levels in the ipNF95.11b C. Reduced CXCL12 may limit chemotaxis and the recruitment of antiviral immune cells [46], while MASP1 downregulation, given its role as a key serine protease in lectin complement activation, suggests weakened innate antiviral recognition.[38,39] Together with the elevated expression of neural lineage genes, this reduced antiviral signature may create a transcriptional state that blunts early antiviral defenses and cellular susceptibility to ZIKV.

In summary, we show that ZIKV infects NF1-associated PNF and MPNST cells, triggering cytotoxic and inflammatory responses that underscore its therapeutic potential. We acknowledge that our use of four cell lines does not fully reflect the genetic diversity of NF1, and that these *in vitro* findings require future *in vivo* validation to address systemic toxicities and evaluate the therapeutic index of ZIKV. Nevertheless, the fact that ZIKV effectively infected an NF1 primary culture is highly promising, suggesting that primary models

could serve as rapid tools to predict tumor susceptibility before treatment. Overall, our findings advance the field of oncolytic virotherapy and highlight ZIKV as a compelling candidate for NF1-derived tumors.

MATERIALS AND METHODS

ZIKV strain and viral detection

The ZIKVBR 17 strain (GenBank KU497555), kindly provided by Dr. Pedro Vasconcelos (Evandro Chagas Institute, Belém, Brazil), was propagated in serum-free Vero cells. Viral titers (PFU/mL) were quantified by plaque assays. Vero cells (ATCC CCL-81) were seeded at 7.5×10^4 cells per well in 24-well plates and incubated at 37°C with 5% CO_2 for 48 h. Supernatants collected 48 h after infecting NF1 tumor cell lines with ZIKV at MOIs of 0.1, 1, and 5 were serially diluted and applied to Vero cells for 1 h. Following adsorption, cells were overlaid with a 1:1 mixture of culture medium and 0.6% agarose and incubated for 96 h. Cells were then fixed with 3.7% paraformaldehyde for 40 min, overlays removed, and monolayers stained with crystal violet to visualize plaques.

Human NF1-associated PNF and MPNST tumor cell lines

Four human NF1-associated tumor Schwann cell lines (referred to as NF1 tumor cell lines) were used in this study: three derived from PNF (ipNF95.11b C, ipNF04.4, and ipNF95.6) and one from an MPNST (sNF02.2), generously provided by Dr. Margareth Wallace at the University of Florida, USA.[40] All cell lines were maintained in Dulbecco's modified Eagle medium/nutrient mixture F12 (DMEM/F12; Gibco) with 10% fetal bovine serum (FBS, Gibco) and 1% antibiotic-antimycotic (Anti-anti; Gibco) at 37°C in a humidified atmosphere with 5% CO2.

3D spheroid culture of NF1 tumor cell lines

Cells (1×10^4 per well) were seeded in DMEM/F12 with 10% FBS in ultra-low-attachment 96-well round-bottom plates (Corning, 7007) and centrifuged at 300 $\times g$ for 3 min. Spheroids were incubated at 37°C with 5% CO_2 for up to 3 days. Then, 100 μL of medium was removed and replaced with 50 μL of ZIKV at MOIs of 0.1, 1, or 5. After 1 h, 100 μL of complete medium was added, and cultures were maintained for 72 h. Spheroid size was assessed by optical microscopy (EVOS XL) and measured using ImageJ.

Cell viability assays for 2D and 3D cell cultures of NF1 tumor cell lines

For 2D cultures, 2×10^4 cells per well were seeded in 96-well plates and incubated for 24 h, then infected with ZIKV at MOIs of 0.1, 1, or 5 for 1 h. After replacing the inoculum with fresh medium, cell viability was measured at 72 hpi with the MTT assay (3- [4,5- dimethylthiazol-2-yl]-2,5-diphenyl tetrazolium bromide). Cells received 100 μL of 1 mg mL^{-1} MTT (Sigma-Aldrich) for 3 h at 37°C, the solution was removed, and 150 μL ethanol was added to dissolve formazan. Absorbance at 570 nm was recorded, and viability was expressed relative to untreated controls.

For 3D cultures, viability was assessed by live/dead staining. Spheroids were incubated with 100 μL of calcein AM (2 μM) and ethidium homodimer-1 (4 μM) for 30 min, washed with PBS, and imaged under an inverted fluorescence microscope (Carl Zeiss). Metabolic activity was quantified using the PrestoBlue assay (Invitrogen); 10% reagent was added per well and incubated for 1 h at 37°C, and fluorescence was measured at 560/590 nm on a Synergy HT reader (BioTek).

Monitoring cell growth after ZIKV infection using real-time cell analysis

Briefly, 3×10^4 cells per well were seeded into E-Plate 96 (Agilent) and incubated for 24 h before infection with ZIKV at MOIs of 0.1, 1, or 5 for 1 h. After replacing the inoculum with fresh medium, real-time cell growth was monitored using the xCELLigence RTCA system (Agilent) at 37°C with 5% CO_2. The E-Plate's gold microelectrodes measure impedance, expressed as the cell index (CI). Impedance readings were collected every 15 min for 7 days and analyzed using RTCA software (v.2.0).

Cell lysates and western blotting

Total protein was extracted using RIPA buffer (Thermo Scientific) with protease inhibitors. Lysates were cleared by centrifugation (15,000 rpm, 20 min, 4°C), and protein levels were quantified using the BCA assay (Thermo Fisher). 30μg of protein were resolved on 4%–12% SDS-PAGE (Bio-Rad) and transferred to polyvinylidene fluoride (PVDF) membranes. Membranes were washed, blocked with 5% BSA, and incubated overnight at 4°C with primary antibodies against AXL (CST 8661), ITGAV (Abcam ab124968), and β-actin (CST 13700). After incubation with HRP-conjugated secondary antibodies for 1 h, bands were visualized using the ChemiDoc system (Bio-Rad).

Immunofluorescence staining

For immunofluorescence, cells were fixed in 4% paraformaldehyde for 15 min, permeabilized with 0.2% Triton X-100 for 30 min, and blocked with 5% BSA for 1 h. Primary antibodies included anti-NS2B (GeneTex), anti-vimentin and anti-NS1 (Invitrogen), anti-Ki67 (Abcam), anti-SOX10 and anti-SOX2 (Invitrogen), and Alexa Fluor 647-conjugated anti-S100β (Abcam). Alexa Fluor 488 goat anti-rabbit and Alexa Fluor 546 goat anti-mouse secondary antibodies (1:1000) were applied for 2 h. Nuclei were counterstained with DAPI, and images were acquired using a Zeiss LSM 510 confocal microscope.

Flow cytometry

Flow cytometry was used to quantify apoptotic and necrotic cells with the CellEvent Caspase-3/7 Green Assay (Thermo Fisher). Cells (1×10^6 per well) were seeded in 6-well plates, incubated for 24 h, and infected with ZIKV (MOCK, MOI 0.1, 1, or 5) for 1 h before continuing culture for 72 h. Cells were then harvested, washed, and incubated with the caspase-3/7 reagent for 30 min; SYTOX AADvanced was added during the final 5 min. Samples were analyzed on a BD Symphony A1 flow cytometer.

Determination of cytokines and chemokines

Cytokine and chemokine levels were quantified using the MILLIPLEX MA Human Cytokine/Chemokine Magnetic Bead Panel (Merck Millipore) on the Luminex® MAGPIX platform. Cluster heatmaps and PCA were generated with SRplot.[41] Protein-protein interaction networks were analyzed using STRING v.12[42] with a high-confidence interaction threshold of 0.70.

Establishment of patient-derived primary cell cultures harboring NF1 mutation (C30322)

Neurofibroma fragments were obtained from a volunteer patient under a protocol approved by the institutional research ethics committee (CAAE: 20271719.8.0000.0065). These samples were used to generate primary cell cultures for subsequent ZIKV infection. Briefly, tumor fragments were mechanically sectioned into small pieces and maintained in DMEM/F12 (Gibco) supplemented with 10% FBS (Maxpec, Thermo Fisher Scientific) and 1% antibiotic-antimycotic solution (Gibco), at 37°C in a humidified atmosphere containing 5% CO_2 (Figure S1).

Next-generation sequencing and variants filtering

Single-nucleotide variants and indels were annotated and filtered using VarSeq v.1.8 (Golden Helix). Variants were required to meet quality thresholds (coverage $\geq$ 20, Phred $\geq$ 20, VAF $\geq$ 0.05) and were excluded if common (gnomAD v.4.1.0 GroupMax AF $\geq$ 0.01). Retained variants included missense changes with REVEL $\geq$ 0.64,[43] and LoF variants in COSMIC Cancer Gene Census genes.[44] Pathogenicity was assessed using ACMG/AMP guidelines and ClinVar,[45] keeping only pathogenic or likely pathogenic calls. All final variants were manually reviewed in IGV to remove sequencing artifacts.[45]

RNA extraction and sequencing

Total RNA was extracted with the RNeasy Mini Kit (Qiagen). RNA concentration was measured by NanoDrop, and quality assessed with an Agilent Bioanalyzer. Sequencing yielded a mean on-target coverage of 57.6×, with 94.6% of bases covered by >10 reads. Reads were aligned with HISAT2,[46] and differential expression was analyzed using edgeR[47] with Benjamini-Hochberg false discovery rate (FDR) correction.[48] Sample relationships were evaluated by PCA and Pearson correlation. CellMarker 2024 enrichment was performed using clusterProfiler, assigning Gene Ontology (GO) terms for molecular function, cellular component, and biological process. GO terms were considered significant at $p \leq 0.05$ and $q \leq 0.05$.

Real-time qPCR

Total RNA was isolated from tumor tissues using Qiazol lysis reagent (Qiagen) and purified with the RNeasy Mini Kit (Qiagen). cDNA was synthesized from 500 ng of RNA in a 20 μL reaction using the High-Capacity cDNA Reverse Transcription Kit (Applied Biosystems). qPCR was performed on a QuantStudio 5 Real-Time PCR System (Thermo Fisher Scientific) with Fast SYBR Green Master Mix (Invitrogen). Primers are listed in Table S1.

Statistical analysis

The results are reported as means ± standard deviations (SDs). Data were statistically analyzed with two-way ANOVA test followed by Tukey's post hoc test in GraphPad Prism (v.10). Statistical significance was considered at $^{*}p < 0.05$, $^{**}p < 0.01$, $^{***}p < 0.001$, and $^{****}p < 0.0001$.

DATA AND CODE AVAILABILITY

The data that support the findings of this study are available from the corresponding author upon request.

ACKNOWLEDGMENTS

The authors thank Professor Margaret Wallace from the University of Florida for providing the NF1 cell lines and for her constructive feedback. This study was supported by the São Paulo Research Foundation (FAPESP, grant nos. 2013/08028-1 and 2014/50931-3) and by INCT (Instituto Nacional de Ciência e Tecnologia, grant no. 465355/2014). The authors also acknowledge the fellowships from FAPESP granted to R.O.F. (2020/14109-8) and H.L.M. (2022/02212-4). The authors further acknowledge the technical and scientific assistance of the Human Genome and Stem Cell Research Center team.

AUTHOR CONTRIBUTIONS

R.O.F., conceptualization, data curation, formal analysis, investigation, methodology, project administration, validation, visualization, writing – original draft, and writing – review and editing; H.L.M., conceptualization, data curation, formal analysis, investigation, methodology, project administration, validation, visualization, writing – original draft, and writing – review and editing; E.H.F.J., investigation and methodology; I.G., formal analysis, investigation, and methodology; C.C.A., formal analysis and software; G.B.d.C., formal analysis and methodology; A.A., formal analysis, investigation, and methodology; G.S., data curation and formal analysis; R.B.P., investigation, methodology, and supervision; R.S.M.-N., formal analysis and validation; R.S., formal analysis and validation; H.M., formal analysis and methodology; O.K.O., funding acquisition, methodology, resources, and supervision; K.S.C., data curation, funding acquisition, methodology, supervision, and writing – original draft; M.Z., conceptualization, funding acquisition, investigation, methodology, project administration, supervision, visualization, and writing – original draft.

DECLARATION OF INTERESTS

M.Z. and O.K.O. are co-founders of Vyro Biotherapeutics.

SUPPLEMENTAL INFORMATION

Supplemental information can be found online at https://doi.org/10.1016/j.omton.2026.201370.

A video abstract is available at https://doi.org/10.1016/j.omton.2026.201370#mmc2.

REFERENCES

1. Korfhage, J., and Lombard, D.B. (2019). Malignant Peripheral Nerve Sheath Tumors: From Epigenome to Bedside. Mol. Cancer Res. *17*, 1417–1428. https://doi.org/10.1158/1541-7786.MCR-19-0147.

2. Boyd, K.P., Korf, B.R., and Theos, A. (2009). Neurofibromatosis type 1. J. Am. Acad. Dermatol. *61*, 1–16. https://doi.org/10.1016/j.jaad.2008.12.051.

3. Rad, E., and Tee, A.R. (2016). Neurofibromatosis type 1: Fundamental insights into cell signalling and cancer. Semin. Cell Dev. Biol. *52*, 39–46. https://doi.org/10.1016/j.semcdb.2016.02.007.

4. Giraud, J.-S., Bièche, I., Pasmant, É., and Tlemsani, C. (2023). NF1 alterations in cancers: therapeutic implications in precision medicine. Expert Opin. Investig. Drugs *32*, 941–957. https://doi.org/10.1080/13543784.2023.2263836.

5. Anastasaki, C., Orozco, P., and Gutmann, D.H. (2022). RAS and beyond: the many faces of the neurofibromatosis type 1 protein. Dis. Model. Mech. *15*, dmm049362. https://doi.org/10.1242/dmm.049362.

6. Anderson, J.L., and Gutmann, D.H. (2015). Neurofibromatosis type 1. Handb. Clin. Neurol. *132*, 75–86. https://doi.org/10.1016/B978-0-444-62702-5.00004-4.

7. Ge, L.-L., Xing, M.-Y., Zhang, H.-B., and Wang, Z.-C. (2022). Neurofibroma development in neurofibromatosis type 1: insights from cellular origin and Schwann cell lineage development. Cancers *14*, 4513. https://doi.org/10.3390/cancers14184513.

8. Fisher, M.J., Blakeley, J.O., Weiss, B.D., Dombi, E., Ahlawat, S., Akshintala, S., Belzberg, A.J., Bornhorst, M., Bredella, M.A., Cai, W., et al. (2022). Management of neurofibromatosis type 1-associated plexiform neurofibromas. Neuro. Oncol. *24*, 1827–1844. https://doi.org/10.1093/neuonc/noac146.

9. Moertel, C.L., Hirbe, A.C., Shuhaiber, H.H., Viskochil, D., Sidhu, A., Bielamowicz, K.J., Weber, M., Li, J., Smith, L.M., Weintraub, L., et al. (2024). ReNeu: A pivotal phase 2b trial of mirdametinib in children and adults with neurofibromatosis type 1 (NF1)-associated symptomatic inoperable plexiform neurofibroma (PN). J. Clin. Oncol. *42*, 3016. 3016-3016. https://doi.org/10.1200/jco.2024.42.16_suppl.3016.

10. Hoy, S.M. (2025). Mirdametinib: First Approval. Drugs *85*, 977–984. https://doi.org/10.1007/s40265-025-02190-0.

11. Armstrong, A.E., Belzberg, A.J., Crawford, J.R., Hirbe, A.C., and Wang, Z.J. (2023). Treatment decisions and the use of MEK inhibitors for children with neurofibromatosis type 1-related plexiform neurofibromas. BMC cancer *23*, 553. https://doi.org/10.1186/s12885-023-10996-y.

12. Khan, S., Alson, D., Sun, L., Maloney, C., and Sun, D. (2024). Leveraging Neural Crest-Derived Tumors to Identify NF1 Cancer Stem Cell Signatures. Cancers *16*, 3639. https://doi.org/10.3390/cancers16213639.

13. Su, K.Y., and Balasubramaniam, V.R.M.T. (2019). Zika virus as oncolytic therapy for brain cancer: Myth or Reality? Front. Microbiol. *10*, 2715. https://doi.org/10.3389/fmicb.2019.02715.

14. Ferreira, R.O., Granha, I., Ferreira, R.S., Bueno, H.d.S., Okamoto, O.K., Kaid, C., and Zatz, M. (2021). Effect of Serial Systemic and Intratumoral Injections of Oncolytic ZIKVBR in Mice Bearing Embryonal CNS Tumors. Viruses *13*, 2103. https://doi.org/10.3390/v13102103.

15. Kaid, C., Goulart, E., Caires-Júnior, L.C., Araujo, B.H.S., Soares-Schanoski, A., Bueno, H.M.S., Telles-Silva, K.A., Astray, R.M., Assoni, A.F., Júnior, A.F.R., et al. (2018). Zika Virus Selectively Kills Aggressive Human Embryonal CNS Tumor Cells In Vitro and In Vivo. Cancer Res. *78*, 3363–3374. https://doi.org/10.1158/0008-5472.CAN-17-3201.

16. Tang, H., Hammack, C., Ogden, Sarah C., Wen, Z., Qian, X., Li, Y., Yao, B., Shin, J., Zhang, F., Lee, Emily M., et al. (2016). Zika Virus Infects Human Cortical Neural Progenitors and Attenuates Their Growth. Cell Stem Cell *18*, 587–590. https://doi.org/10.1016/j.stem.2016.02.016.

17. Kaid, C., Madi, R.A.D.S., Astray, R., Goulart, E., Caires-Junior, L.C., Mitsugi, T.G., Moreno, A.C.R., Castro-Amarante, M.F., Pereira, L.R., Porchia, B.F.M.M., et al. (2020). Safety, tumor reduction, and clinical impact of Zika virus injection in dogs with advanced-stage brain tumors. Mol. Ther. *28*, 1276–1286. https://doi.org/10.1016/j.ymthe.2020.03.004.

18. Crane, A.T., Chrostek, M.R., Krishna, V.D., Shiao, M., Toman, N.G., Pearce, C.M., Tran, S.K., Sipe, C.J., Guo, W., Voth, J.P., et al. (2020). Zika virus-based immunotherapy enhances long-term survival of rodents with brain tumors through upregulation of memory T-cells. PloS one *15*, e0232858. https://doi.org/10.1371/journal.pone.0232858.

19. Jouhilahti, E.-M., Peltonen, S., Heape, A.M., and Peltonen, J. (2011). The Pathoetiology of Neurofibromatosis 1. Am. J. Pathol. *178*, 1932–1939. https://doi.org/10.1016/j.ajpath.2010.12.056.

20. Ling, B.C., Wu, J., Miller, S.J., Monk, K.R., Shamekh, R., Rizvi, T.A., DeCourten-Myers, G., Vogel, K.S., DeClue, J.E., and Ratner, N. (2005). Role for the epidermal growth factor receptor in neurofibromatosis-related peripheral nerve tumorigenesis. Cancer Cell *7*, 65–75. https://doi.org/10.1016/j.ccr.2004.10.016.

21. Sun, X., and Kaufman, P.D. (2018). Ki-67: more than a proliferation marker. Chromosoma *127*, 175–186. https://doi.org/10.1007/s00412-018-0659-8.

22. Büttner, T., Wang, X., Krishnacoumar, B., Papadamakis, A., Cicek, S., Schild, Y., Winning, S., Littwitz-Salomon, E., Ehrhardt, A., Dittmer, U., et al. (2026). Spheroids reveal hypoxia-driven spatial restriction of adenoviral infection. Sci. Rep. *16*, 15864. https://doi.org/10.1038/s41598-026-53319-4.

23. Ma, H.L., Garcia, L.S.A., Ishiba, R., Dametto, L.C., da Silva Moraes, K.A., Oliveira Ferreira, R., Okamoto, O.K., and Zatz, M. (2026). Modeling glioblastoma in 3D hydrogels enables investigation of Zika virus targeting and immune modulation in oncolytic virotherapy. Biomater. Adv. *179*, 214492. https://doi.org/10.1016/j.bioadv.2025.214492.

24. Robles-Minutti, J.N., Cuapa-González, M.A., Márquez-Domínguez, L., Arenas-Hernández, M.M.P., and Santos-López, G. (2025). Unlocking the Gate: Keys to Understanding Zika Virus Interactions with Human Cell Receptors. Curr. Trop. Med. Rep. *12*, 13. https://doi.org/10.1007/s40475-025-00347-1.

25. Calderón-Peláez, M.-A., Maradei Anaya, S., Bedoya-Rodríguez, I., González-Ipuz, K., Vera-Palacios, D., Buitrago, I., Castellanos, J., and Velandia-Romero, M. (2024). Zika Virus: A Neurotropic Warrior against High-Grade Gliomas—Unveiling Its Potential for Oncolytic Virotherapy. Viruses *16*, 561. https://doi.org/10.3390/v16040561.

26. Xu, J., Liu, C., An, Y., Sun, J., Wang, S., and Xia, Q. (2025). Mechanisms of Oncolytic Virus-Induced Multi-Modal Cell Death and Therapeutic Prospects. Int. J. Mol. Sci. *26*, 9770. https://doi.org/10.3390/ijms26199770.

27. Allani, M., Tiwari, V., and Tiwari, V. (2024). Caspase-driven cancer therapies: Navigating the bridge between lab discoveries and clinical applications. Cell Biochem. Funct. *42*, e3944. https://doi.org/10.1002/cbf.3944.

28. Frost, H.K., Kodama, A., Ekström, P., and Dahlin, L.B. (2016). G-CSF prevents caspase 3 activation in Schwann cells after sciatic nerve transection, but does not improve nerve regeneration. Neuroscience *334*, 55–63. https://doi.org/10.1016/j.neuroscience.2016.07.045.

29. Souza, B.S.F., Sampaio, G.L.A., Pereira, C.S., Campos, G.S., Sardi, S.I., Freitas, L.A.R., Figueira, C.P., Paredes, B.D., Nonaka, C.K.V., Azevedo, C.M., et al. (2016). Zika virus infection induces mitosis abnormalities and apoptotic cell death of human neural progenitor cells. Sci. Rep. *6*, 39775. https://doi.org/10.1038/srep39775.

30. Salem, M.L., Salman, S., and Barnawi, I.O. (2021). Brief in vitro IL-12 conditioning of CD8+ T Cells for anticancer adoptive T cell therapy. Cancer Immunol. Immunother. *70*, 2751–2759. https://doi.org/10.1007/s00262-021-02887-7.

31. Terrén, I., Orrantia, A., Mosteiro, A., Vitallé, J., Zenarruzabeitia, O., and Borrego, F. (2021). Metabolic changes of Interleukin-12/15/18-stimulated human NK cells. Sci. Rep. *11*, 6472. https://doi.org/10.1038/s41598-021-85960-6.

32. Wang, M., Chen, S., He, X., Yuan, Y., and Wei, X. (2024). Targeting inflammation as cancer therapy. J. Hematol. Oncol. *17*, 13. https://doi.org/10.1186/s13045-024-01528-7.

33. Chen, L., Zhou, C., Chen, Q., Shang, J., Liu, Z., Guo, Y., Li, C., Wang, H., Ye, Q., Li, X., et al. (2022). Oncolytic Zika virus promotes intratumoral T cell infiltration and improves immunotherapy efficacy in glioblastoma. Mol. Ther. Oncolytics *24*, 522–534. https://doi.org/10.1016/j.omto.2022.01.011.

34. Sherwood, M., Mitsugi, T.G., Kaid, C., Coke, B., Zatz, M., Maringer, K., Okamoto, O.K., and Ewing, R.M. (2025). Multi-omics analysis reveals key immunogenic signatures induced by oncolytic Zika virus infection of paediatric brain tumour cells. Sci. Rep. *15*, 13090. https://doi.org/10.1038/s41598-025-97804-8.

35. Zhou, C., Chen, Q., Chen, Y., and Qin, C.-F. (2023). Oncolytic zika virus: new option for glioblastoma treatment. DNA Cell Biol. *42*, 267–273. https://doi.org/10.1089/dna.2022.0375.

36. Mo, J., Anastasaki, C., Chen, Z., Shipman, T., Papke, J., Yin, K., Gutmann, D.H., and Le, L.Q. (2021). Humanized neurofibroma model from induced pluripotent stem cells delineates tumor pathogenesis and developmental origins. J. Clin. Invest. *131*, e139807. https://doi.org/10.1172/JCI139807.

37. Finzsch, M., Schreiner, S., Kichko, T., Reeh, P., Tamm, E.R., Bösl, M.R., Meijer, D., and Wegner, M. (2010). Sox10 is required for Schwann cell identity and progression beyond the immature Schwann cell stage. J. Cell Biol. *189*, 701–712. https://doi.org/10.1083/jcb.200912142.

38. Teicher, B.A., and Fricker, S.P. (2010). CXCL12 (SDF-1)/CXCR4 Pathway in Cancer. Clin. Cancer Res. *16*, 2927–2931. https://doi.org/10.1158/1078-0432.CCR-09-2329.

39. Matsushita, M., Endo, Y., and Fujita, T. (1998). MASP1 (MBL-Associated Serine Protease 1). Immunobiology *199*, 340–347. https://doi.org/10.1016/S0171-2985(98)80038-7.

40. Li, H., Chang, L.-J., Neubauer, D.R., Muir, D.F., and Wallace, M.R. (2016). Immortalization of human normal and NF1 neurofibroma Schwann cells. Lab. Invest. *96*, 1105–1115. https://doi.org/10.1038/labinvest.2016.88.

41. Tang, D., Chen, M., Huang, X., Zhang, G., Zeng, L., Zhang, G., Wu, S., and Wang, Y. (2023). SRplot: A free online platform for data visualization and graphing. PloS one *18*, e0294236. https://doi.org/10.1371/journal.pone.0294236.

42. Szklarczyk, D., Kirsch, R., Koutrouli, M., Nastou, K., Mehryary, F., Hachilif, R., Gable, A.L., Fang, T., Doncheva, N.T., Pyysalo, S., et al. (2023). The STRING database in 2023: protein–protein association networks and functional enrichment analyses for any sequenced genome of interest. Nucleic Acids Res. *51*, D638–D646. https://doi.org/10.1093/nar/gkac1000.

43. Ioannidis, N.M., Rothstein, J.H., Pejaver, V., Middha, S., McDonnell, S.K., Baheti, S., Musolf, A., Li, Q., Holzinger, E., Karyadi, D., et al. (2016). REVEL: An Ensemble Method for Predicting the Pathogenicity of Rare Missense Variants. Am. J. Hum. Genet. *99*, 877–885. https://doi.org/10.1016/j.ajhg.2016.08.016.

44. Sondka, Z., Bamford, S., Cole, C.G., Ward, S.A., Dunham, I., and Forbes, S.A. (2018). The COSMIC Cancer Gene Census: describing genetic dysfunction across all human cancers. Nat. Rev. Cancer *18*, 696–705. https://doi.org/10.1038/s41568-018-0060-1.

45. Robinson, J.T., Thorvaldsdóttir, H., Wenger, A.M., Zehir, A., and Mesirov, J.P. (2017). Variant Review with the Integrative Genomics Viewer. Cancer Res. *77*, e31–e34. https://doi.org/10.1158/0008-5472.CAN-17-0337.

46. Mortazavi, A., Williams, B.A., McCue, K., Schaeffer, L., and Wold, B. (2008). Mapping and quantifying mammalian transcriptomes by RNA-Seq. Nat. Methods *5*, 621–628. https://doi.org/10.1038/nmeth.1226.

47. Robinson, M.D., McCarthy, D.J., and Smyth, G.K. (2010). edgeR: a Bioconductor package for differential expression analysis of digital gene expression data. Bioinformatics *26*, 139–140. https://doi.org/10.1093/bioinformatics/btp616.

48. Benjamini, Y., and Hochberg, Y. (1995). Controlling the False Discovery Rate: A Practical and Powerful Approach to Multiple Testing. Journal of the Royal Statistical Society Series B: Statistical Methodology *57*, 289–300. https://doi.org/10.1111/j.2517-6161.1995.tb02031.x.

OMTON, Volume *34*

Supplemental information

Therapeutic potential of oncolytic Zika virus in NF1-associated tumors

Raiane Oliveira Ferreira, Hui Ling Ma, Elisa Helena Farias Jandrey, Isabela Granha, Camila Correia Avelino, Gabriel Bandeira do Carmo, Amanda Assoni, Gustavo Sartorelli, Rafael Braga Petito, Rodrigo S. Moura-Neto, Rosane Silva, Hamilton Matushita, Oswaldo Keith Okamoto, Karin Soares Cunha, and Mayana Zatz

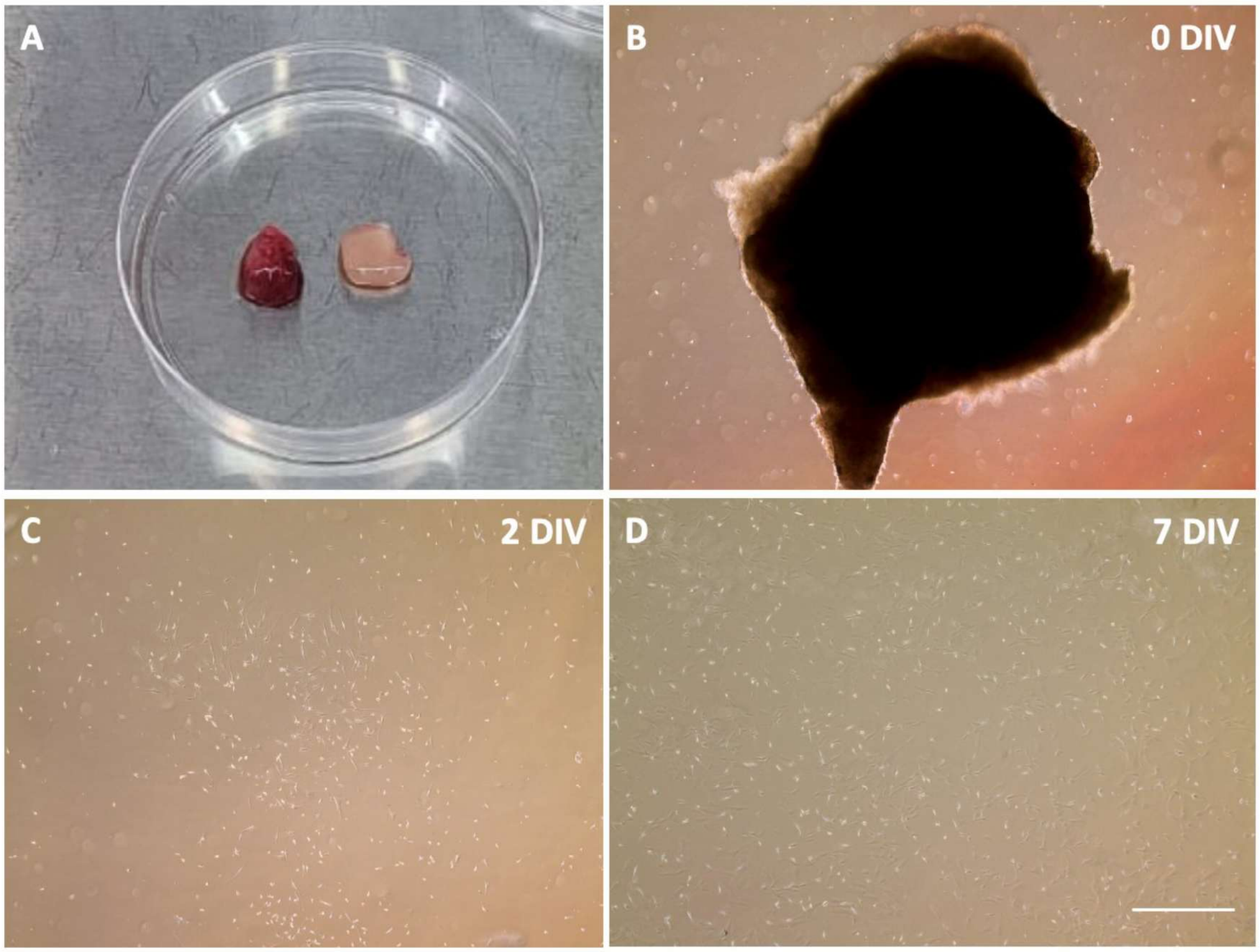

Figure S1. Cell isolation from patient-derived sample. A) Patient-derived fresh tumor tissue. Representative bright-field microscopy of the primary monolayer culture at 0 days *in vitro* (DIV) (B), 2 DIV (C), and 7 DIV (D). Scale bar: 200um.

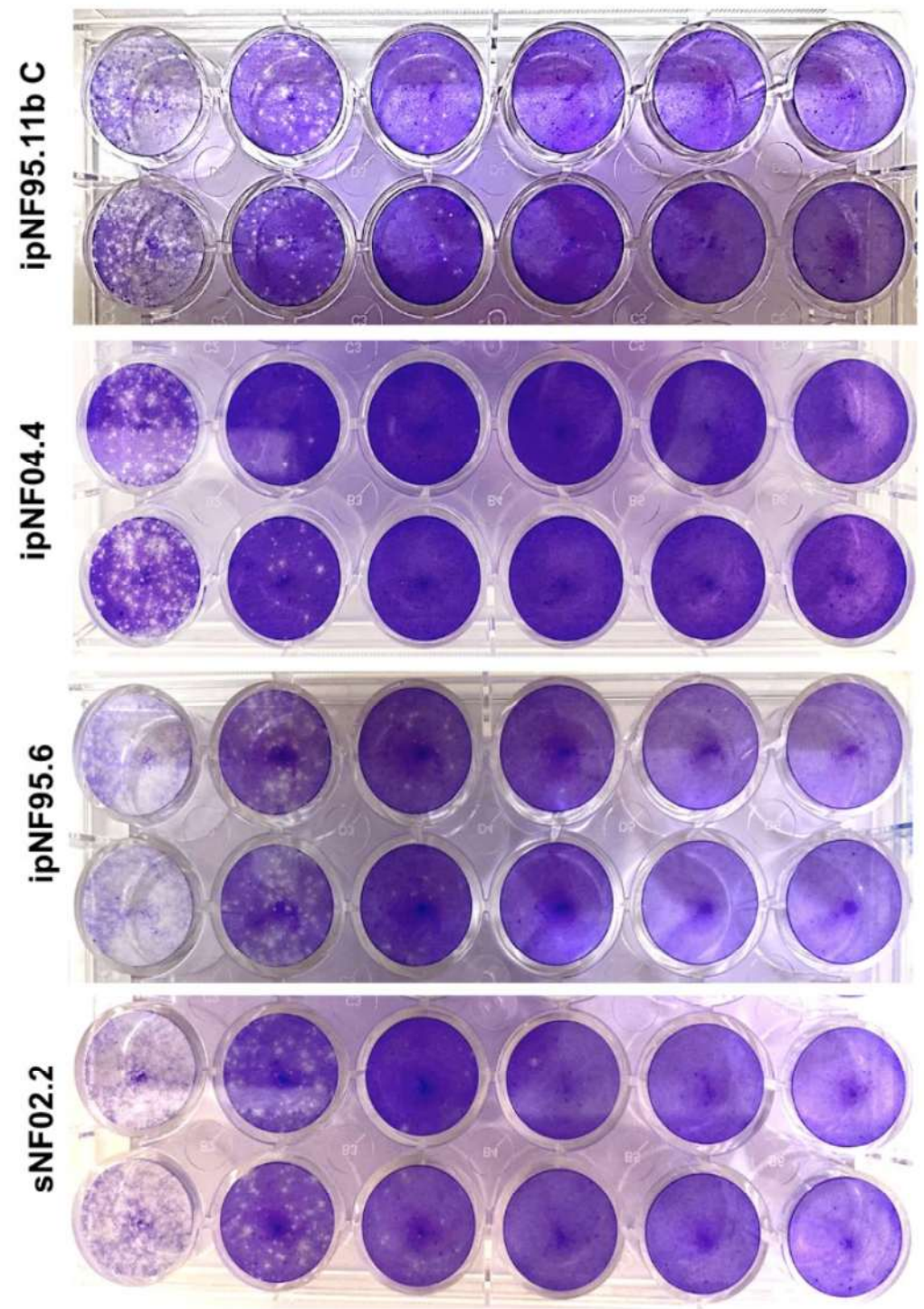

Figure S2. ZIKV replication in NF1 cells. To assess viral replication, PFU assays were measured in the supernatants collected from NF1 cells infected with ZIKV at MOI of 5, 48 h post-infection. A) Viral plaques were visualized and counted in VERO cells four days after infection. B) Quantification of infectious viral particles is expressed as Log PFU mL^{-1}. Significance was considered at *$p < 0.05$, **$p < 0.01$, ***$p < 0.001$, ****$p < 0.001$ and ns = not significant.

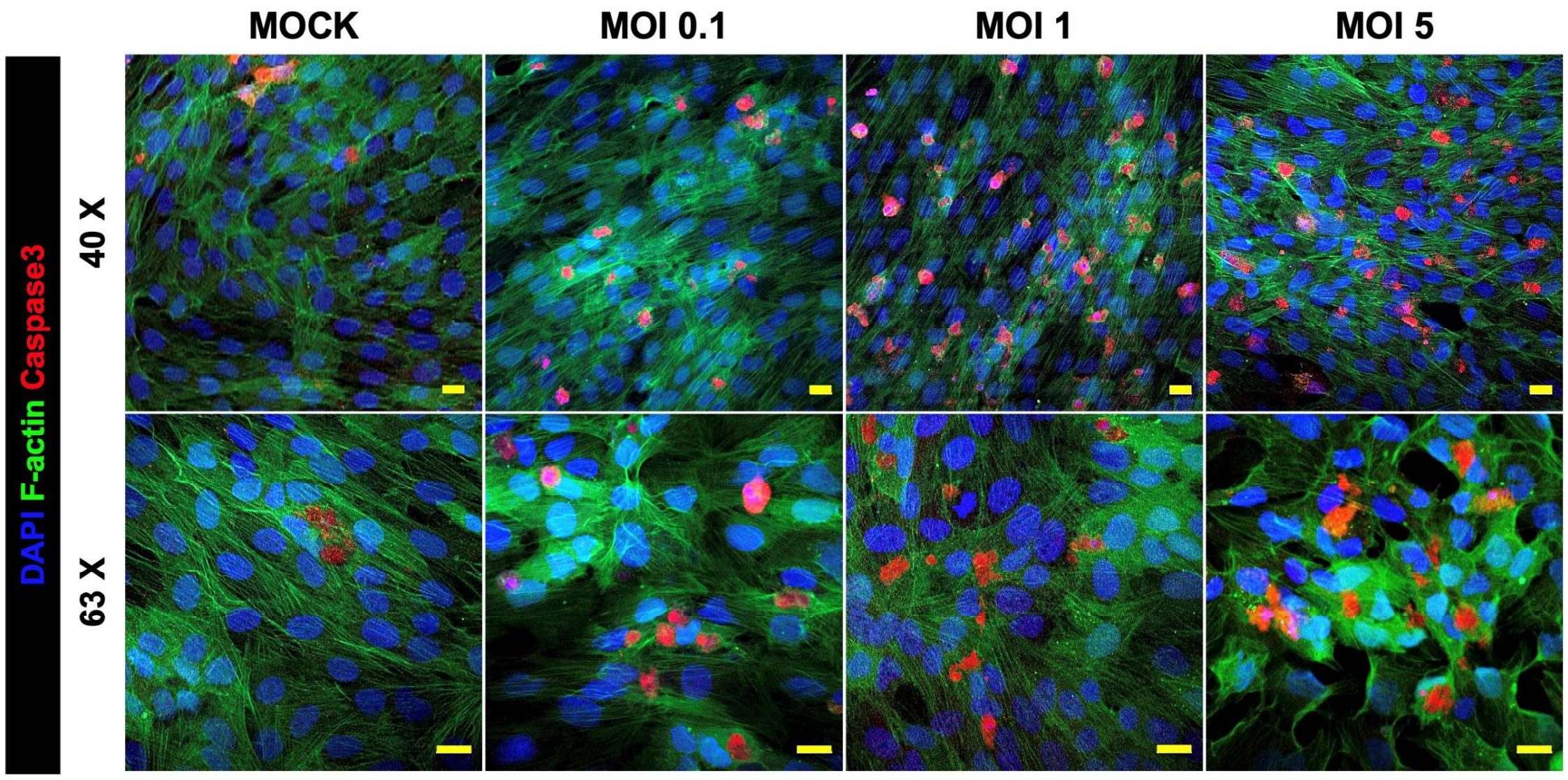

Figure S3. Immunofluorescence images of ipNF95.11b C 72hpi for clivage-caspase3 (red) and F-actin (green). Scale bar: 20um.

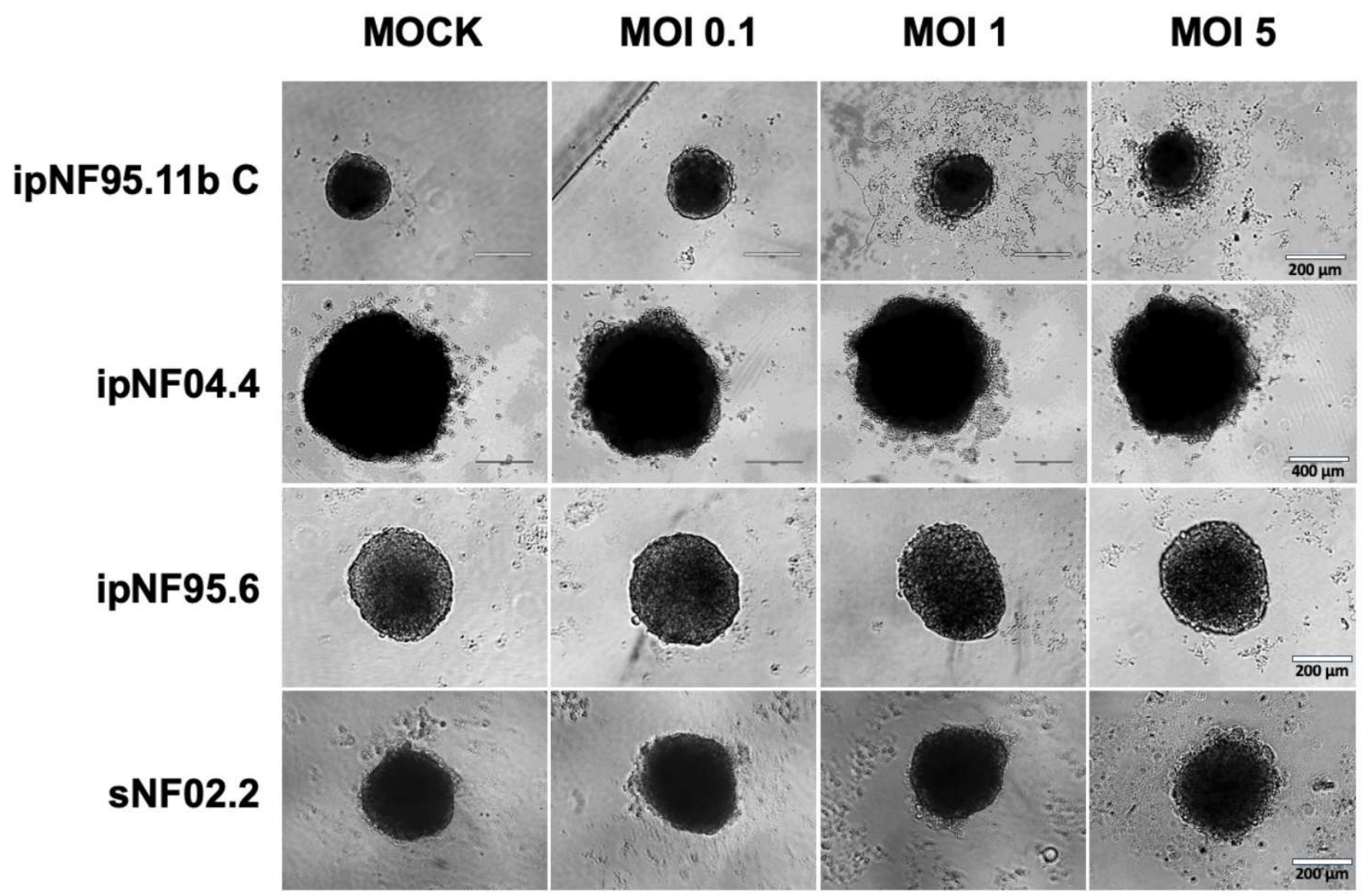

Figure S4. Bright-field images of 3D neurofibromatosis spheroid cultures, infected with ZIKV at MOCK, MOI 0.1, 1, and 5, were captured 72 hpi, revealing alterations in spheroid morphology and diameter changes.

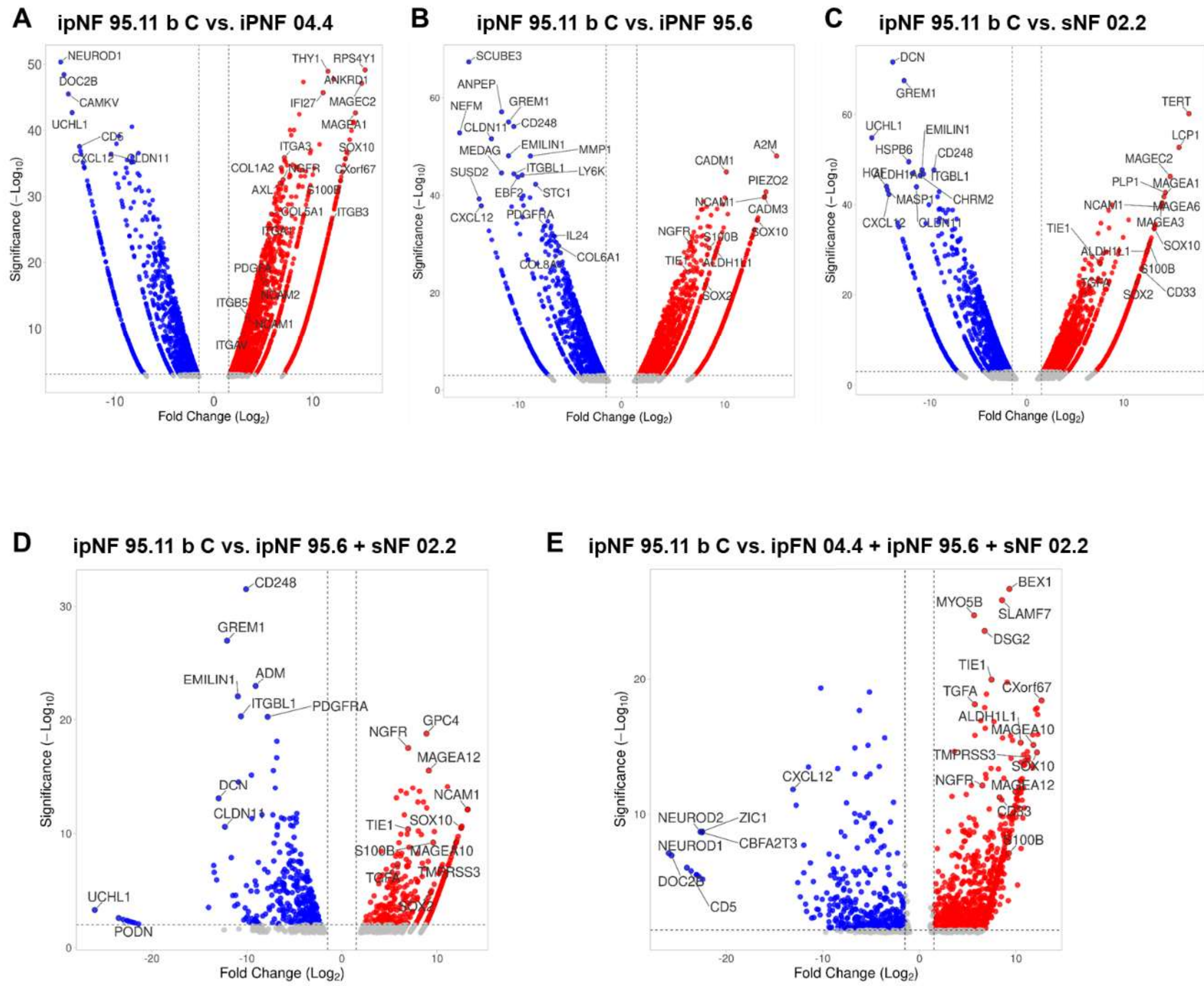

Figure S5. Differential gene-expression profiles across NF1 tumor cell line comparisons. Volcano plots depict $\log_2$ fold-change (x-axis) versus statistical significance ($-\log_{10}$ p-value, y-axis). Upregulated transcripts are shown in red, and downregulated transcripts in blue. Individual represent pairwise contrasts relative to the ipNF 95.11 b C cell line: A) vs. ipNF 04.4. B) vs. ipNF 95.6. C) vs. sNF 02.2. D) vs. ipNF 95.6 + sNF 02.2. E) vs. ipNF 04.4 + ipNF 95.6 + sNF 02.2.

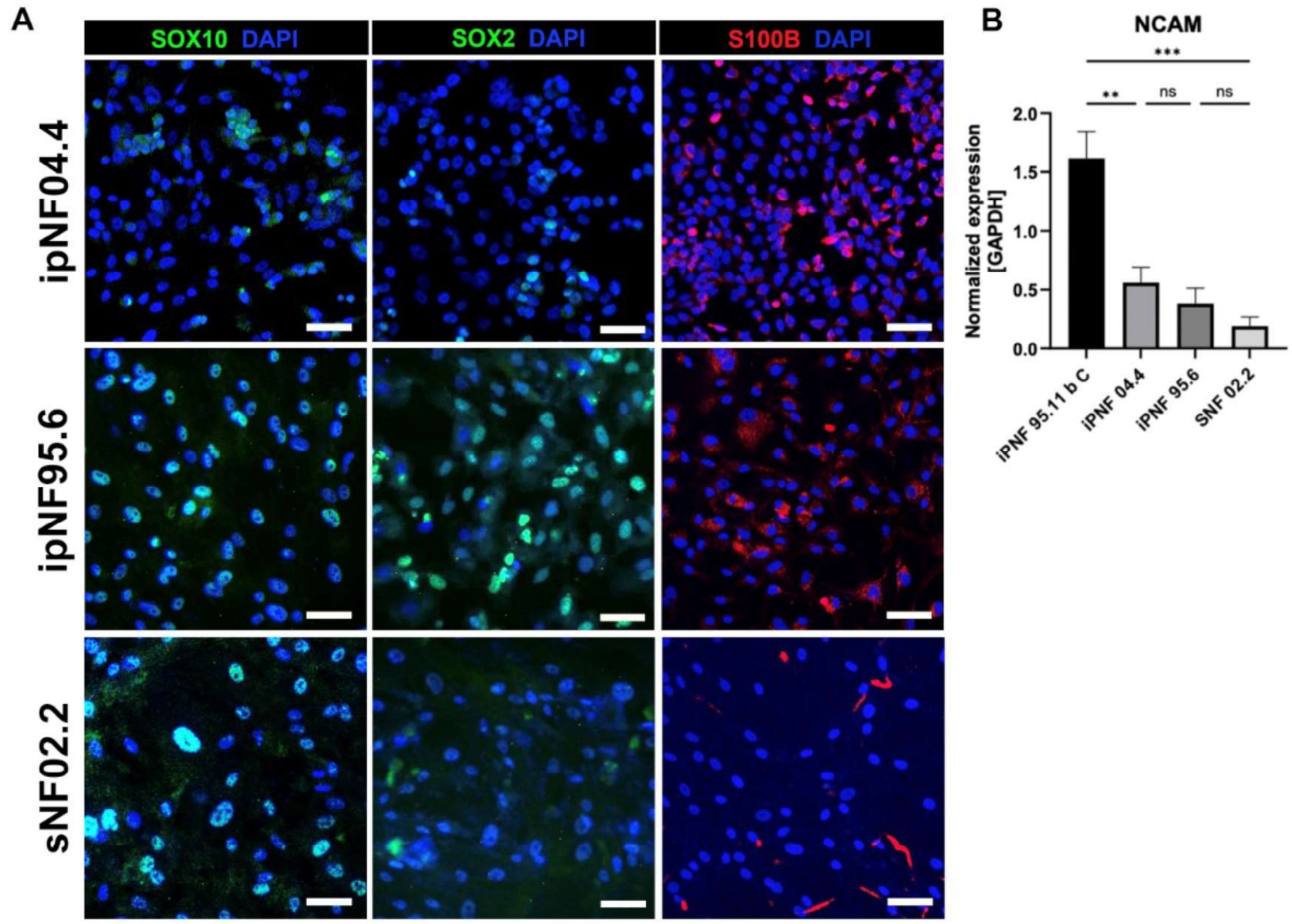

Figure S6. A) Immunofluorescence images of NF1 tumor cell lines for SOX10 (green), SOX2 (green), S100B (red), and nuclei (DAPI, blue). B) Real-time PCR results showing the relative expression levels of NCAM in NF1 tumor cell lines, normalized to GAPDH. Statistical significance: $^{**}p < 0.01$, $^{***}p < 0.001$, ns = not significant.

Table S1. Primer sequence for RT-qPCR

Target	Sequence (5'- 3')
**GAPDH**	F: GCTGGAGGGCGAAGAAAAC
	R: GCCTTCTGACACGGATTTGG
**NCAM1**	F: CCGGAGAGTACATCTGCACC
	R: GAGCTTAGGTGCACTGGGTT
**NESTIN**	F: GGC GCA CCT CAA GAT GTC C
	R: CTT GGG GTC CTG AAA GCT
**S100B**	F: AGGAAGAGGATGTCTGAGCTG
	R: CCTCCGGGTTAGGGTCTACA
**SOX10**	F: GTTGACTGTTGCACCCACAC
	R: GAGTGGTAAGGCCTCCGATG
**SOX2**	F: TTCACATGTCCCAGCACTACCAGA
	R: TCACATGTGTGAGAGGGGCAGTGTGC

Table S2. Pathogenic variants identified in whole-exome sequencing analysis.

Cell line	Sequence Ontology	Germline NF1 mutation
ipNF95.11b C	frameshift_variant	c.1756delACTA (p.Thr586Valfs*18)
ipNF95.6	stop_gained<br>stop_gained	p.Arg816Ter/ p.Arg2237Ter
sNF02.2	stop_gained	p.Arg2237Ter
C30322	frameshift_variant	p.Val2229Leufs*15
